# Prophage-Derived Regions in *Curtobacterium* Genomes: Good Things, Small Packages

**DOI:** 10.3390/ijms24021586

**Published:** 2023-01-13

**Authors:** Peter Evseev, Anna Lukianova, Rashit Tarakanov, Anna Tokmakova, Anastasia Popova, Eugene Kulikov, Mikhail Shneider, Alexander Ignatov, Konstantin Miroshnikov

**Affiliations:** 1 Shemyakin-Ovchinnikov Institute of Bioorganic Chemistry, Russian Academy of Sciences, Miklukho-Maklaya Str., 117997 Moscow, Russia; 2 Department of Plant Protection, Russian State Agrarian University—Moscow Timiryazev Agricultural Academy, Timiryazevskaya Str. 49, 127434 Moscow, Russia; 3 School of Biological and Medical Physics, Moscow Institute of Physics and Technology National Research University, Institutskiy Per, 9, 141701 Dolgoprudny, Russia; 4 State Research Center for Applied Microbiology and Biotechnology, 142279 Obolensk, Russia; 5 Research Center of Biotechnology, Winogradsky Institute of Microbiology, Russian Academy of Sciences, Prosp. 60-letia Oktyabrya, 7-2, 117312 Moscow, Russia; 6 Agrobiotechnology Department, Agrarian and Technological Institute, RUDN University, Miklukho-Maklaya Str. 6, 117198 Moscow, Russia

**Keywords:** viral genomics, analysis of genomic data, *Curtobacterium*, phages, prophages, endolysins, glycan depolymerases

## Abstract

*Curtobacterium* is a genus of Gram-positive bacteria within the order *Actinomycetales*. Some *Curtobacterium* species (*C. flaccumfaciens*, *C. plantarum*) are harmful pathogens of agricultural crops such as soybean, dry beans, peas, sugar beet and beetroot, which occur throughout the world. Bacteriophages (bacterial viruses) are considered to be potential curative agents to control the spread of harmful bacteria. Temperate bacteriophages integrate their genomes into bacterial chromosomes (prophages), sometimes substantially influencing bacterial lifestyle and pathogenicity. About 200 publicly available genomes of *Curtobacterium* species, including environmental metagenomic sequences, were inspected for the presence of sequences of possible prophage origin using bioinformatic methods. The comparison of the search results with several ubiquitous bacterial groups showed the relatively low level of the presence of prophage traces in *Curtobacterium* genomes. Genomic and phylogenetic analyses were undertaken for the evaluation of the evolutionary and taxonomic positioning of predicted prophages. The analyses indicated the relatedness of *Curtobacterium* prophage-derived sequences with temperate actinophages of siphoviral morphology. In most cases, the predicted prophages can represent novel phage taxa not described previously. One of the predicted temperate phages was induced from the *Curtobacterium* genome. Bioinformatic analysis of the modelled proteins encoded in prophage-derived regions led to the discovery of some 100 putative glycopolymer-degrading enzymes that contained enzymatic domains with predicted cell-wall- and cell-envelope-degrading activity; these included glycosidases and peptidases. These proteins can be considered for the experimental design of new antibacterials against *Curtobacterium* phytopathogens.

## 1. Introduction

Bacteria belonging to the genus *Curtobacterium* are of great interest. These actinomycetes have been found in many of Earth’s microbiomes. In spite of being unable to form spores, *Curtobacterium* spp. are nevertheless abundant in soil [1,2], marine sediments [3] and in the air up to the stratosphere [4]. *Curtobacteria* demonstrate enhanced tolerance to drought, salinity, UV irradiation and metal ions, and have been suggested to play an important role in plant adaptation to stress conditions [5,6,7]. Being typical inhabitants of the rhizosphere of many plants, *Curtobacterium* spp. have been shown to promote seed germination [8,9] and plant growth [10,11], and to suppress the growth of phytopathogens [12,13]. A pathovar, *Curtobacterium flaccumfaciens* pv. *flaccumfaciens*, is an economically important plant pathogen [14] and, occasionally, a human pathogen [15,16]. *Curtobacterium* spp. are often found in plant microbiomes relevant to bacterial diseases [17], and may promote the development of pathogenic fungi [18].

Genetic determinants of environmental adaptation and plant pathogenicity among *Curtobacterium* sp. are not completely understood [19], and may be influenced by mobile elements such as plasmids as well as prophages or their remnants. Bacteriophages are viruses that infect bacteria. Lytic or virulent phages reproduce, lyse the cell and release progeny phages upon infecting bacteria. Lysogenic or temperate phages may multiply via the lytic cycle or adopt a latent state in the cell. Prophages are comprised of phage DNA in the integrated state. Fully functional prophages are capable of excising from the bacterial chromosome, either spontaneously or in response to specific signals; they then lyse the host cells at some subsequent generation upon induction [20]. As a result of mutations, prophages can lose the ability for induction, but still play a role in bacterial adaptation, introducing new fitness factors for the host cell [21,22,23].

Prophages can constitute as much as 10–20% of a bacterial genome and contribute to interstrain variability. Prophages may harbour virulence factors and pathogenicity islands, thereby playing an important role in the emergence of pathogens [24,25]. The purpose of the present study was to ascertain the repertoire, and possible role, of prophage sequences in the genomes of *Curtobacterium* sp., to propose the origin and phylogeny of initial phages, considering recent changes in bacteriophage taxonomy, and to reveal and systematise information about cytolytic enzymes encoded in prophage sequences which may have biotechnological potential.

## 2. Results

### 2.1. Predicted Prophages in Curtobacterial and Other Bacterial Genomes

In early summer 2022, genomes of 197 strains assigned to the genus *Curtobacterium* were identified in the NCBI Genome Database [26]. Thirty-six strains were classified as *C. flaccumfaciens* and 28 strains were classified as pathovars of *C. flaccumfaciens*. Previous research [19] revealed inconsistencies in the taxonomy of genus *Curtobacterium* and called for taxonomic re-evaluation. Thus, all 197 genomes have been used for analyses.

The search for genomic regions of prophage origin has been performed using the online server PHASTER [27] and the pipeline PhiSpy [28]. PHASTER currently seems to be the most widely used prophage prediction tool [29], and a recently published comparative study demonstrated the high levels of accuracy, precision, recall and f_1_ score of PhiSpy [30]. PHASTER has identified 161 possible prophage regions, while only one of them has been defined as an intact phage; PhiSpy has found 75 prophage regions. The most substantial part of similar predicted prophages referred to 32 genomic loci containing the genes, encoding UDP-N-acetylglucosamine transferase, N-acetylglucosaminyl-diphospho-decaprenol L-rhamnosyltransferase, teichoic acid biosynthesis protein F, dTDP-4-dehydrorhamnose 3,5-epimerase, glycosyltransferase of PMT family, undecaprenyl-phosphate 4-deoxy-4-formamido-L-arabinose transferase, 4-amino-4-deoxy-L-arabinose transferase or related glycosyltransferase, dTDP-glucose 4,6-dehydratase (RmlB) and dTDP-4-dehydrorhamnose reductase (RfbD), according to the results of PHASTER annotation, BLAST [31] and HHpred [32] searches. These enzymes participate in producing cell wall polysaccharides (CWPSs) and exopolysaccharide (EPS) in Gram-positive bacteria, and lipopolysaccharides in Gram-negative bacteria [33,34,35]. Some genes encoding enzymes for CWPS and EPS could have been acquired via horizontal gene transfer [35]. Several predicted prophages have not contained phage structural genes, but have been shown to possess genes typical for plasmids and other mobile elements such as transposases and conjugative transfer genes [36]. Thus, the results of these bioinformatic tools need additional post-processing, but they can be useful for the assessment of the level of mobile elements’ presence in bacterial genomes.

**Figure 1:**
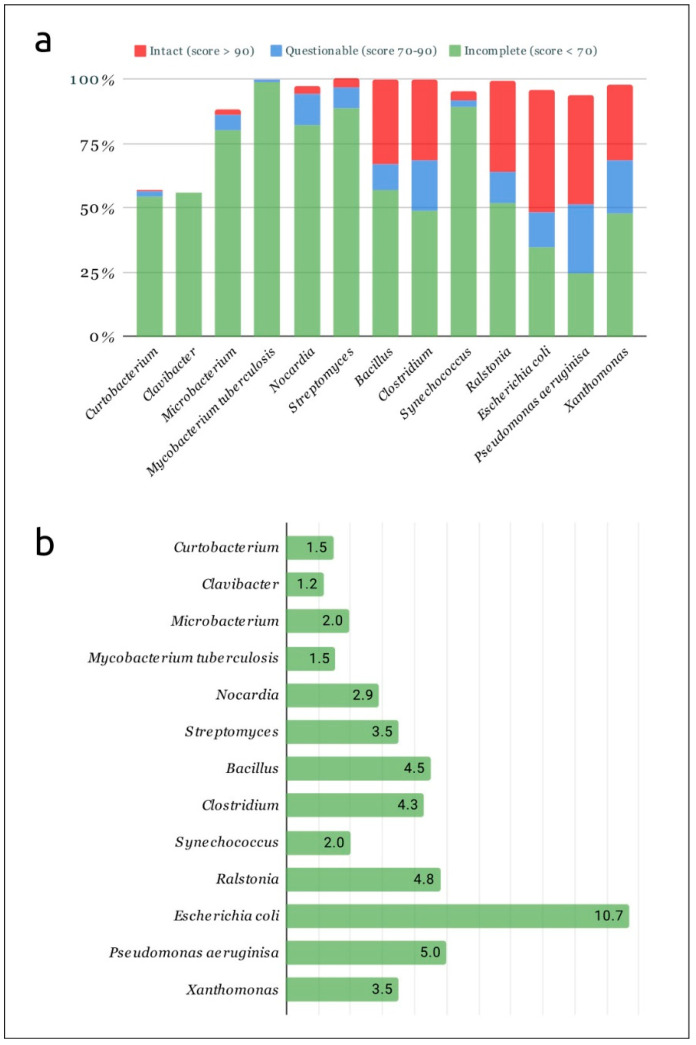
(**a**) Percentage of genomes of different analysed taxonomic groups where PHASTER predicted the presence of prophages. Scoring was carried out according to the PHASTER criteria [37]. (**b**) Average number of prophages per genome predicted with PHASTER, where the prophages were detected.

To estimate the comparative level of saturation of genomes of different bacterial groups with mobile elements, 12 additional groups of pathogenic bacteria, including phytopathogens, were analysed using PHASTER. Each of these groups included 100 complete and draft genomes belonging to genera *Bacillus*, *Clavibacter*, *Clostridium*, *Microbacterium*, *Nocardia*, *Ralstonia*, *Streptomyces*, *Synechococcus* and *Xanthomonas*, and species *Escherichia coli*, *Mycobacterium tuberculosis* and *Pseudomonas aeruginosa*. The results of PHASTER analysis indicated a smaller number of predicted prophage-derived regions in genus *Curtobacterium* and closely related genus *Clavibacter*, compared with other taxa (Figure 1).

### 2.2. Post-Processing of Phaster and PhiSpy Results

**Figure 2:**
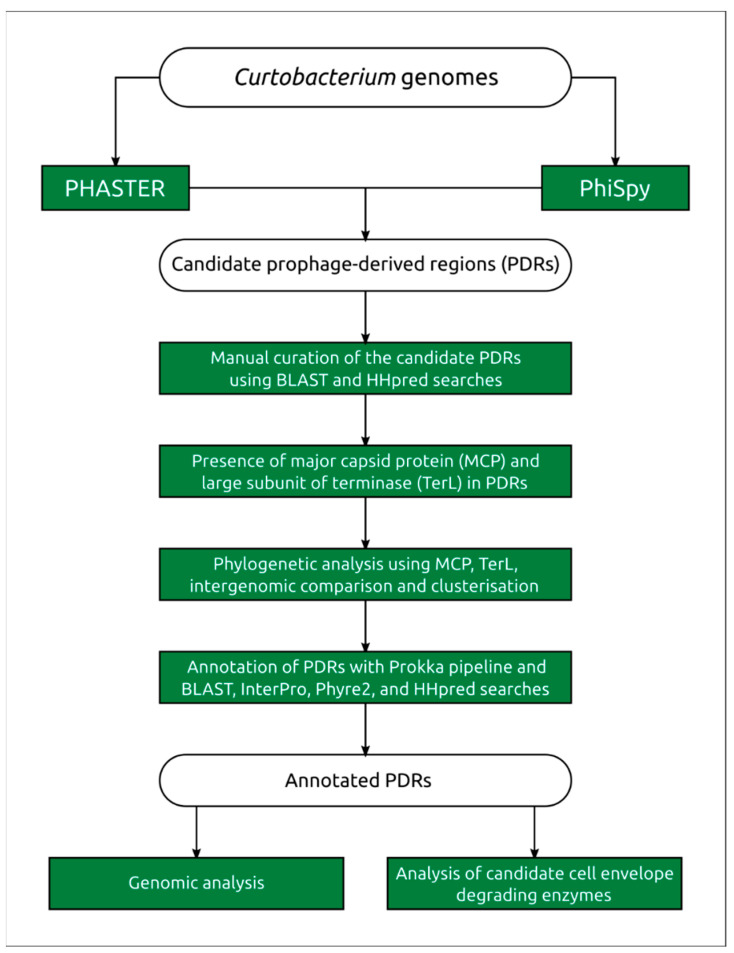
Flow chart of the identification and characterisation of *Curtobacterium* prophage-derived regions. The rectangles represent the tools and methods used for identification of PDRs; the rounded rectangles represent the data used for the analyses.

Predictions by PHASTER and PhiSpy often yielded similar overlapping genomic regions, but manual inspection of putative prophage-derived regions (PDRs) revealed some discrepancies in the predictions of these two tools, and possible inaccuracies in the definition of the borders of the prophage-derived genomic region. PHASTER predicted potential sites for prophage insertion for only 22 putative prophages and the prediction did not look reliable, so the borders of PDRs were suggested on the basis of the phage origin of genes and comparisons with known phage genomes. Post-processing and manual curation of the prediction were conducted in ways similar to those described in [38] (Figure 2).

All of the predicted regions were checked through an analysis of the gene content of predicted regions and their possible prophage origin, with a BLAST search using the NCBI and custom phage databases. The genomic content of these 64 predicted prophage regions was additionally checked through comparisons with genomes of sequenced bacteriophages using an HHpred search. Putative genes of holins and spanins were also checked by the prediction of transmembrane regions. This post-processing revealed 70 prophage-derived regions (PDRs) possessing phage structural genes. Sixty-four regions were found to contain the genes encoding major capsid protein (MCP) and terminase large subunit (TerL). They might represent recently grounded or intact prophages and have been considered for further analysis. General features of these PDRs are listed in Table 1, with their order corresponding to the clustering described below. Genomic regions of putative prophages corresponding to the PDRs C_sp_UCD-KPL2560‖2, C_sp_MCLR17_036‖1, C_luteum_NS184‖1, C_sp_MCBA15_004‖2, C_sp_MCBD17_003‖1, C_luteum_NS184‖1, C_sp_MCBA15_004‖2 and C_sp_MCBD17_003‖1 were trimmed according to the contigs’ limits.

### 2.3. Intergenomic Comparison, Phylogenetic Analysis, Annotations and General Genomic Features

**Figure 3:**
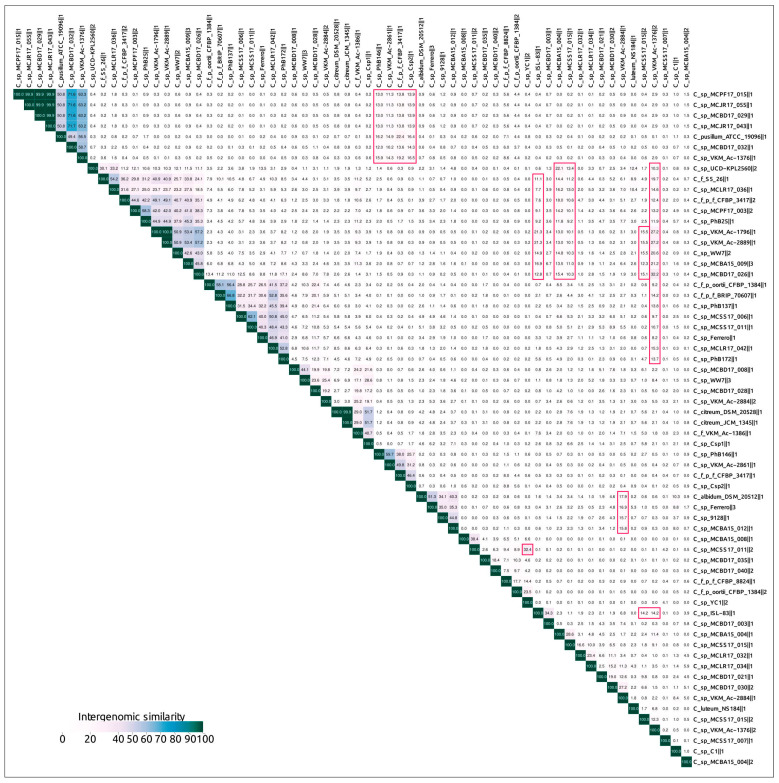
VIRIDIC-generated heatmap of 64 curated PRDs containing major capsid protein and terminase genes. The colour coding indicates the clustering of the phage genomes based on intergenomic similarity. The numbers represent the similarity values for each genome pair, rounded to the first decimal. Examples of heatmap islands indicating similarities between prophages not belonging to main clusters are outlined in red.

Intergenomic comparisons of 64 predicted and curated PDRs conducted using the Virus Intergenomic Distance Calculator (VIRIDIC) [39] (Figure 3) indicated both the relatedness of putative prophages, and a complicated picture of their relationships. Applying the 95% similarity threshold, two groups consisting of two and four PDRs can be considered as the same species. At least 46 PDRs could be grouped in several clusters, but intergenomic distances between different species were higher than the 70% genus cut-off for only two species. This assumes that either most PDRs represent distant taxonomic groups of functional phages, or that these PDRs represent defective prophages, including those inherited vertically before quickly diverging after the loss of the possibility to be induced. Some prophage regions have shown similarities to PDRs belonging to different clusters. This might be the result of genetic mosaicism, featuring phage evolution, especially the evolution of temperate phages [40,41,42]. Thus, clustering of the predicted PDRs using intergenomic similarities can be inconsistent.

**Figure 4:**
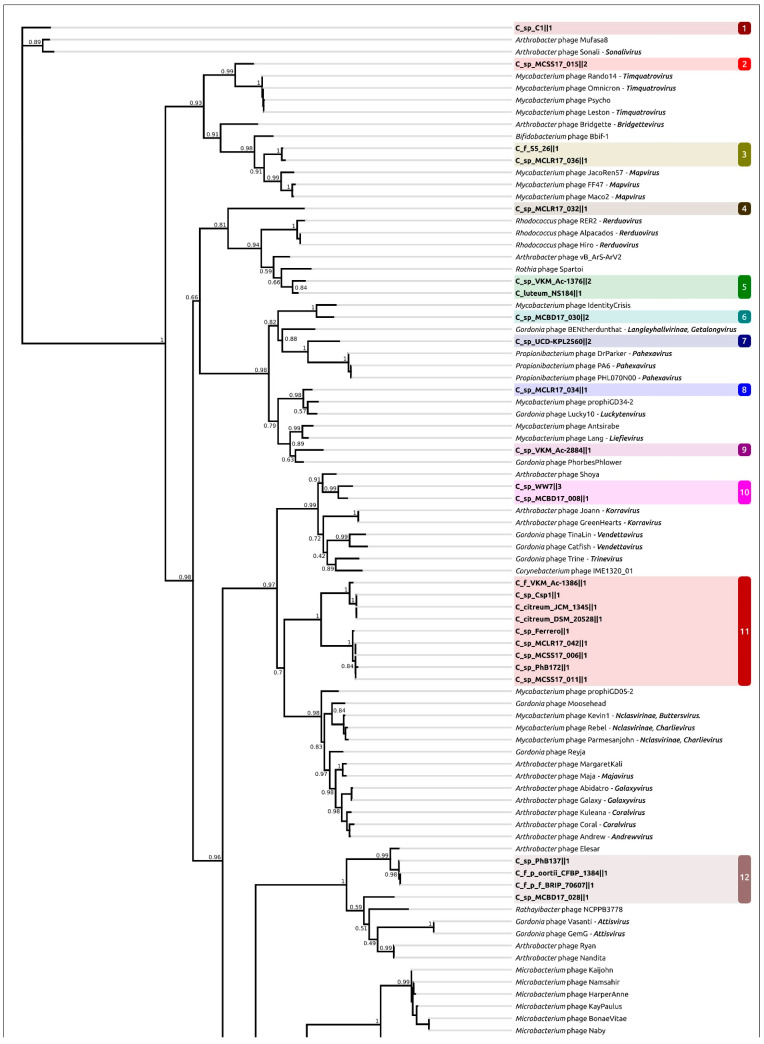

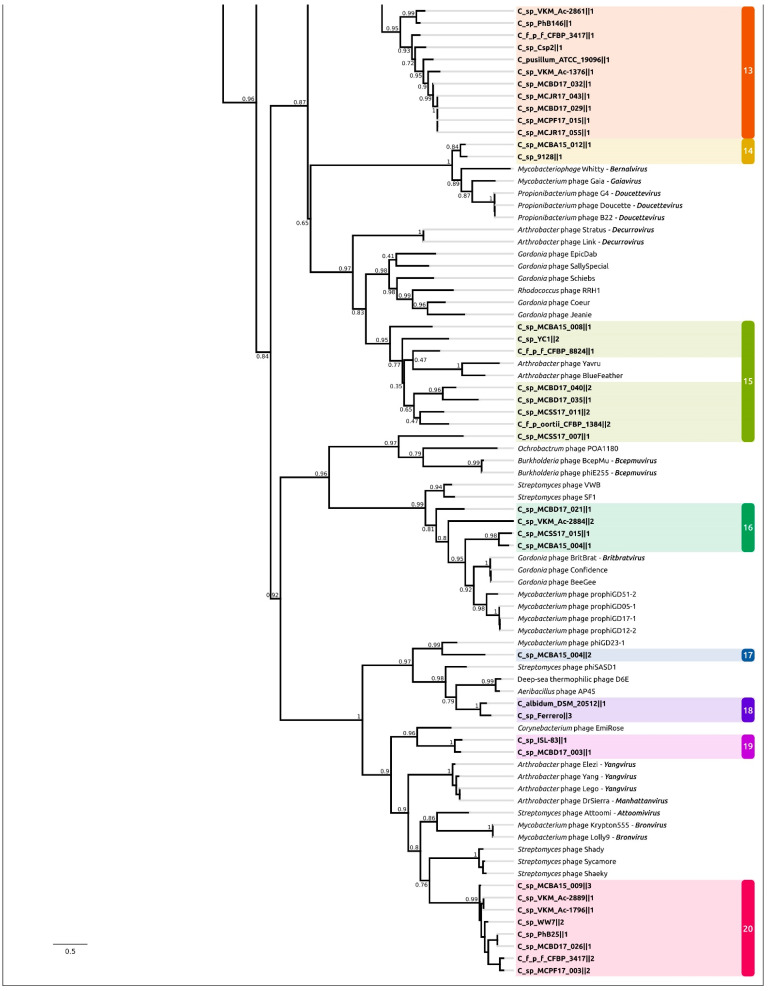
Best-scoring ML phylogenetic tree constructed with 164 amino acid sequences of major capsid protein. The NCBI taxonomy is shown to the right of the phage name. The numbers near the tree branches indicate the TBE values. The total number of bootstrap trees was 1000. The scale bar shows 0.5 estimated substitutions per site and the tree was rooted to C_sp_C1‖1. Different background colours and numbers indicate the clustering proposed for analysis.

Phylogenetic analysis was conducted using the major capsid protein (MCP) and large subunit of terminase (TerL) amino acid sequences encoded in the curated prophage regions and close homologous sequences found with a BLAST search using complete phage genomes available in the NCBI Genome database as of July 2022. The search did not yield the same results for these two proteins, and the topologies of the trees were not identical (Figure 4, Supplementary Figures S1–S3), even though the compositions of clades containing the predicted prophage regions were similar. The dissimilarities might also be the result of the mosaic evolution of phage genomes.

**Figure 5:**
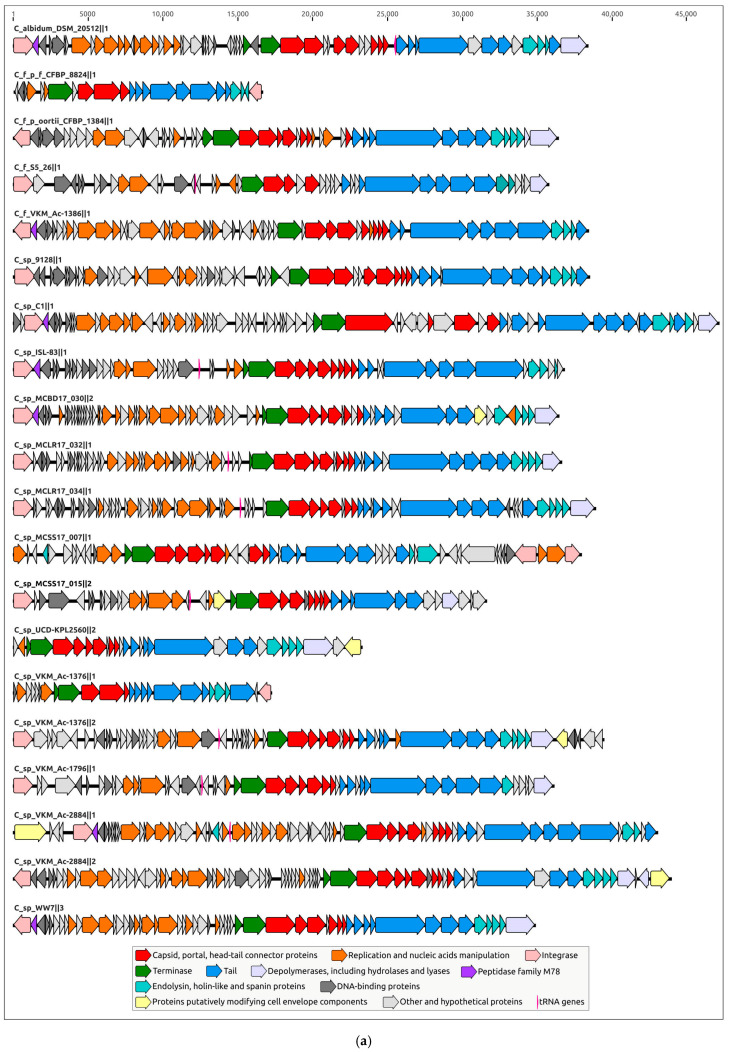

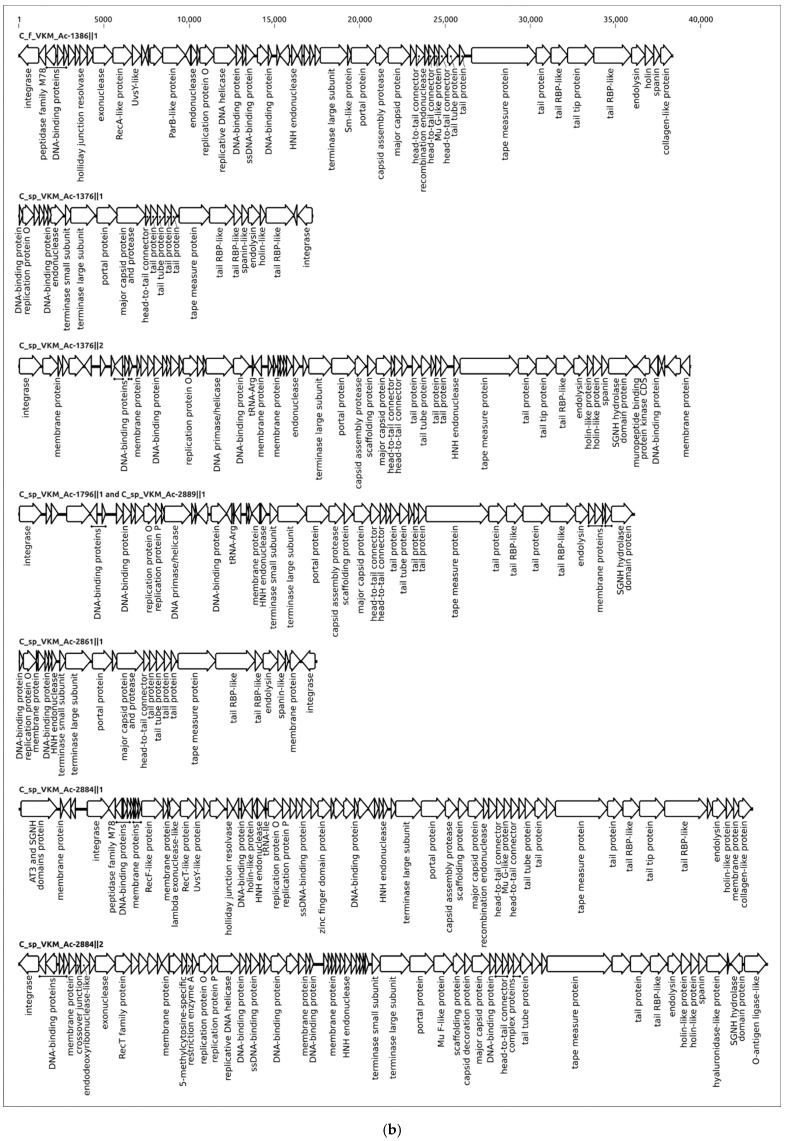
(**a**) Simplified genetic maps of the representatives of each putative prophage cluster shown in Figure 4. (**b**) Genetic maps of seven putative prophages predicted to be contained in *Curtobacterium* genomes of VKM collection strains. Arrows indicate the direction of transcription.

Clustering was performed using the results of MCP phylogenetic analysis. Representatives of each prophage cluster, shown in Figure 4, have been annotated manually using the procedure described in Section 4.2 (Figure 5); the remaining PDRs were annotated using the Prokka pipeline and a custom database constructed with manually annotated PDRs. All PDRs (Supplementary File S1) contained the genes of the tail tape measure protein and did not contain the genes of the tail sheath protein, indicating the siphoviral morphology of corresponding prophages [43]. A total of 197 *Curtobacterium* genomes used for the analyses were also checked for the presence of tail sheath protein homologues using a BLAST search, with the set of tail sheath protein sequences that were used earlier for the characterisation of the evolutionary history of proteins of this class [44]; no such homologues have been found. All PDRs not trimmed by contig limits contained tyrosine integrase genes and DNA-binding protein genes similar to the genes of phage λ lysogeny decision-making proteins Cro and CII. These findings indicate the temperate lifestyle of putative prophages [45]. Interestingly, the groups 1, 6, 9, 10, 11, 18 and 19 (Table 1) contained peptidase family M78 genes neighbouring the integrase genes (Figure 5a). Similar metallo-endopeptidases participate in the regulation of the excision of the ICEBs1 transposon of *Bacillus subtilis*, cleaving the immunity repressor [46].

The replication apparatus of all predicted prophages included the protein similar to the λ replication protein O required for initiation of DNA replication and present in other temperate phages [42,47]. Larger genomes also contained genes similar to another phage λ initiator replication protein P [47]. Most genomes contained DNA primase/helicase genes, as in some lambdoid phages [48]. No DNA polymerase (DNAP) genes were found in the curated PDRs, even though it seemed that, in a few cases, regions of apparently bacterial origin, adjacent to prophages, contained DNAP genes, basically encoding bacterial DNA polymerase III subunits.

Genomic regions of all predicted prophages, not trimmed by contig limits, comprised all other functional modules featuring the tailed phages, including structural and lysis modules. All predicted major capsid proteins featured HK97 fold, typical for *Heunggongvirae* viruses, including tailed bacteriophages [49]. HHpred analysis indicated similarities among the analysed structural proteins and structural proteins of transposable phage Mu [50,51], including Mu F-like and Mu G-like putative capsid assembly proteins (Figure 5b). The lysis cassettes were predicted to encode a three-step lysis system [52] containing the endolysin, holin and spanin genes. The genomes of putative prophages assigned to clusters 1, 4–10, 12 and 17 can contain two adjacent membrane holin-like proteins, possibly possessing the holin–antiholin system, where antiholin controls the timing of host cell lysis by inhibiting holin [53,54]. Most PRDs’ genomes have genes encoding depolymerases of different functionality, as discussed below. They can be released during the lytic stage, facilitating phage escape [55].

The tail modules of analysed RDRs vary in size and complexity and can comprise up to ten or more genes, including the head–tail connector complex genes. Several proteins showed structural similarity to known tail spike proteins and were predicted to contain depolymerase domains. Interestingly, some PDRs can contain the genes that can modify cell envelope components (Figure 5a).

### 2.4. Taxonomy of Related Phages

To define closely related phage taxonomic groups, orthoANI [56] and VIRIDIC tools were used to compare nucleotide sequences of viral genomes. The first step of the analysis included calculations of average nucleotide identity with orthoANI using 20 putative prophage sequences representing 20 clusters (shown in Figure 4) and all 13,477 complete phage genomes available in the NCBI Genome database as of July 2022. Next, 20 putative prophages and the related genomes, found with the orthoANI calculation and having the highest ANI values and average aligned length, were used to estimate intergenomic similarity, using the VIRIDIC tool.

Neither ANI calculations (Supplementary Table S1), nor a VIRIDIC comparison matrix (Supplementary Figure S4), revealed meaningful similarities between *Curtobacterium* PDRs and known complete phage genomes. However, a small likeness of the order of 10% has been detected with some phages infecting *Microbacterium* bacteria, which is phylogenetically close to the genus *Curtobacterium*. In particular, small *Microbacterium* phages with a genome size under 20,000 bp [57], recently assigned to newly established taxa (family *Orlajensenviridae*, subfamily *Pelczarvirinae*, genus *Paopuvirus*) [58], were shown to share distant intergenomic similarities with putative *Curtobacterium* prophages, with a similar genome size, assigned to Group 13.

Phylogenetic analysis using amino acid sequences of conservative proteins can reveal more distant evolutionary and taxonomic relationships [59]. The list of related phage taxonomic groups exposed using the MCP and TerL phylogenies (Figure 4, Supplementary Figure S1) basically includes both unclassified and classified temperate actinophages of siphoviral morphology assigned to subfamily *Nclasvirinae* and several dozen genera not assigned to any subfamily or family, including *Bridgettevirus*, *Britbratvirus, Bronvirus*, *Coralvirus*, *Decurrovirus*, *Fromanvirus*, *Mapvirus*, *Timquatrovirus,* etc. The related phages infect bacteria belonging to genera *Arthrobacter*, *Attisvirus*, *Bifidobacterium*, *Corynebacterium*, *Gordonia*, *Microbacterium*, *Mycobacterium*, *Streptomyces*, *Propionibacterium*, *Rathayibacter* and *Rhodococcus*.

CRISPR spacers represent biological records of past phage–bacteria interactions, and can be used for finding related hosts and phages [60,61]. A CRISPR search found CRISPR repeat regions in only 11 genomes of the 197 *Curtobacterium* genomes analysed, namely in strains *Curtobacterium flaccumcaciens* pv. *flaccumfaciens* (*C. fpf*) BRIP_70615, *C. fpf* BRIP 70615, *C. fpf* CFBP 3417, *C. luteum* JCM 1480, *C. luteum* ATCC 15830, *C. luteum* DSM 20542, *C.* sp. 9128 DE0339, *C.* sp. MCBD17_028, *C.* sp. MCPF17_018, *C.* sp. MCSS17_007 and *C.* sp. WW7. The total number of spacers was 72. BLAST, using the spacers’ sequences, found similar regions, mainly in the genomes of actinophages of siphoviral morphology, including phages belonging to subfamilies *Arquatrovirinae*, *Bclasvirinae*, *Guernseyvirinae*, *Mclasvirinae*, *Nclasvirinae*, *Nymbaxtervirinae*, *Weiservirinae*, unclassified phages and different genera not assigned to subfamilies or families.

### 2.5. Prophage Induction

Six strains of *Curtobacterium* sp. (VKM Ac-2098, VKM Ac-2884, VKM Ac-2861, VKM Ac-1796, VKM Ac-1376 and VKM Ac-2889) were assessed for the presence of inducible prophages through induction using different mitomycin C concentrations, as described. Bioinformatic analysis suggests the presence of prophages integrated into genomes of these strains, and strains were available. The prophage-free *Curtobacterium* strain CFBP 3418 was used as a control for the induction experiments.

It was shown that applying filtrates from bacterial cultures of *Curtobacterium* strains VKM Ac-2098, VKM Ac-2884, VKM Ac-2861, VKM Ac-1796, VKM Ac-1376 and VKM Ac-2889 that were treated with mitomycin C to a final concentration of at least 1 µg/mL resulted in the formation of a lysis zone on the bacterial lawns of all tested *Curtobacterium* sp. strains, except for CFBP 3418 (Supplementary Figure S5). Furthermore, the addition of mitomycin C at a concentration of 1 µg/mL did not lead to growth inhibition of the prophage-free strain CFBP 3418. Thus, this concentration of mitomycin C was chosen as the optimal concentration for prophage induction from bacterial cultures of *Curtobacterium* strains.

**Figure 6:**
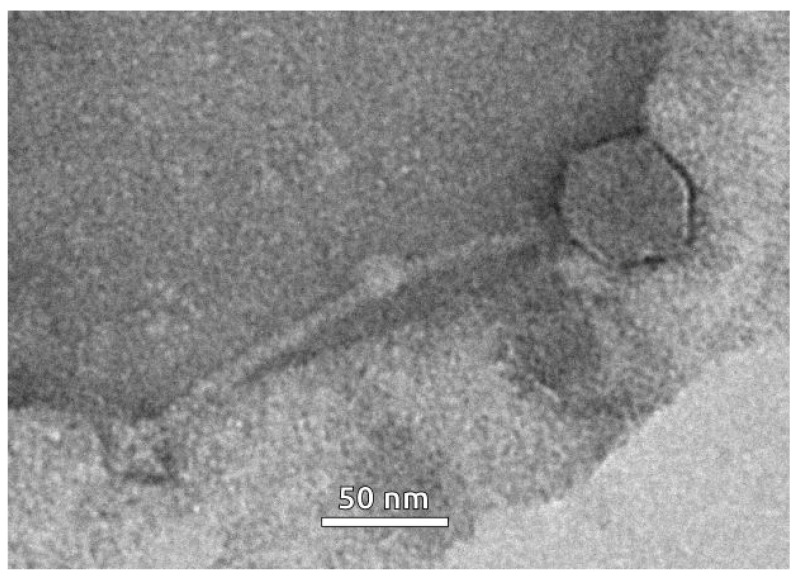
Electron microscopy image of phage particle induced by mitomycin C from the bacterial culture of *Curtobacterium* sp. VKM Ac-2884. The scale bar is 50 nm.

The siphoviral morphology of phage particles induced from the bacterial culture of the *Curtobacterium* strain VKM Ac-2884 was revealed using transmission electron microscopy (TEM) (Figure 6). The induced prophage was characterised with a flexible ~190-nm-long tail and an isometric capsid with a diameter of ~60 nm.

The genome of the strain *Curtobacterium* sp. VKM Ac-2884 was predicted to contain two prophages. Both of them were siphoviruses, and it is impossible to distinguish between them using TEM imaging. A PCR analysis of total DNA isolated from concentrated phage particles after induction was conducted. Amplification was observed only with a set of primers constructed for the detection of phage **C_sp_VKM_Ac-2884‖2** (Supplementary Figure S6).

A phylogenetic analysis using the major capsid protein (Figure 4) indicated the relatedness of phage **C_sp_VKM_Ac-2884‖2** to unclassified phages *Mycobacterium* prophiGD12-2, *Mycobacterium* prophiGD05-1, *Streptomyces* SF1 and *Streptomyces* SF1. The closest classified *Gordonia* phage BritBrat (*Britbratvirus britbrat*) belongs to the *Britbratvirus* genus not assigned to a subfamily or family. The VIRIDIC Intergenomic Distance Calculator failed to indicate any meaningful intergenomic nucleotide similarity between the induced prophage and the related phages listed above. ANI calculations using all phage sequences deposited in NCBI GenBank also failed to find closely related phages with any meaningful average nucleotide identity and coverage. Thus, the induced prophage can represent a new viral genus or a higher-ranked taxon.

### 2.6. Analysis of Phage Endolysins Encoded in PDRs

**Figure 7:**
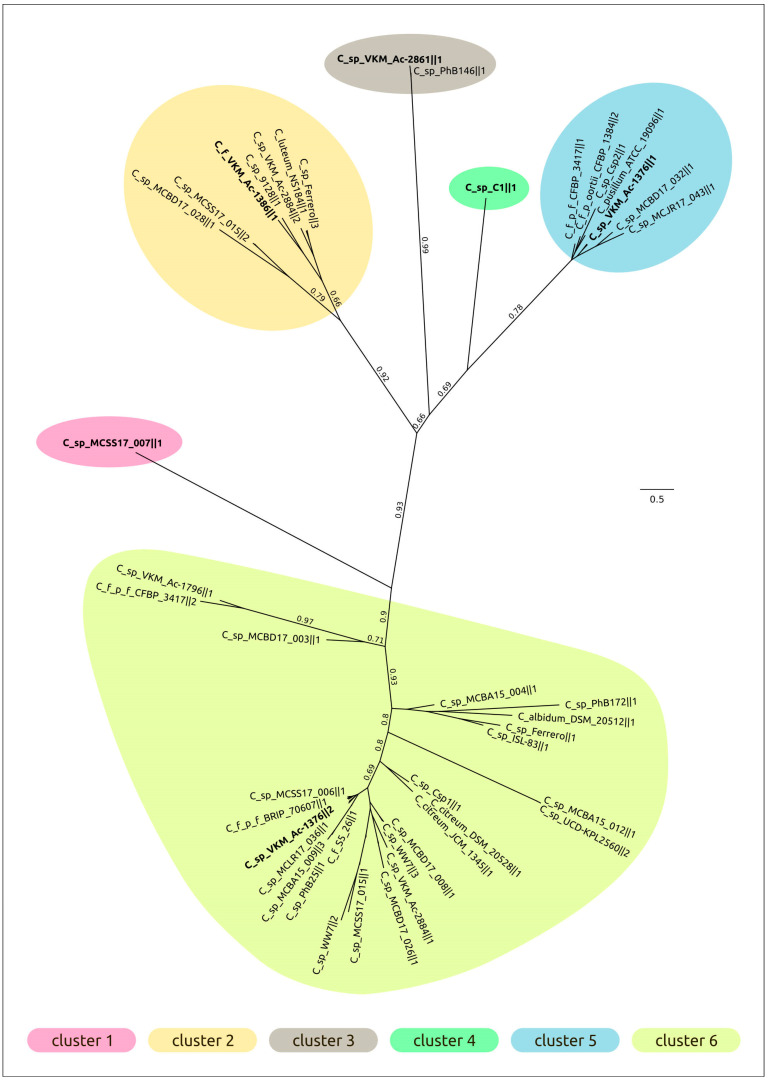
Best-scoring ML phylogenetic tree constructed with 58 amino acid sequences of endolysins. The numbers near the tree branches indicate the TBE values. The total number of bootstrap trees was 1000. The scale bar shows 0.5 estimated substitutions per site and the tree was unrooted. Background colours indicate the clustering proposed for the analysis.

A search for peptidoglycan hydrolase (lysin) genes in the predicted prophage regions indicated the presence of homologues of phage lysins in all PDRs that were not trimmed by contig borders. Fifty-eight lysins found by the search (Supplementary File S2) were clustered using an ML phylogenetic analysis (Figure 7). An HHpred analysis indicated a similar structure and domain architecture within the clusters.

**Figure 8:**
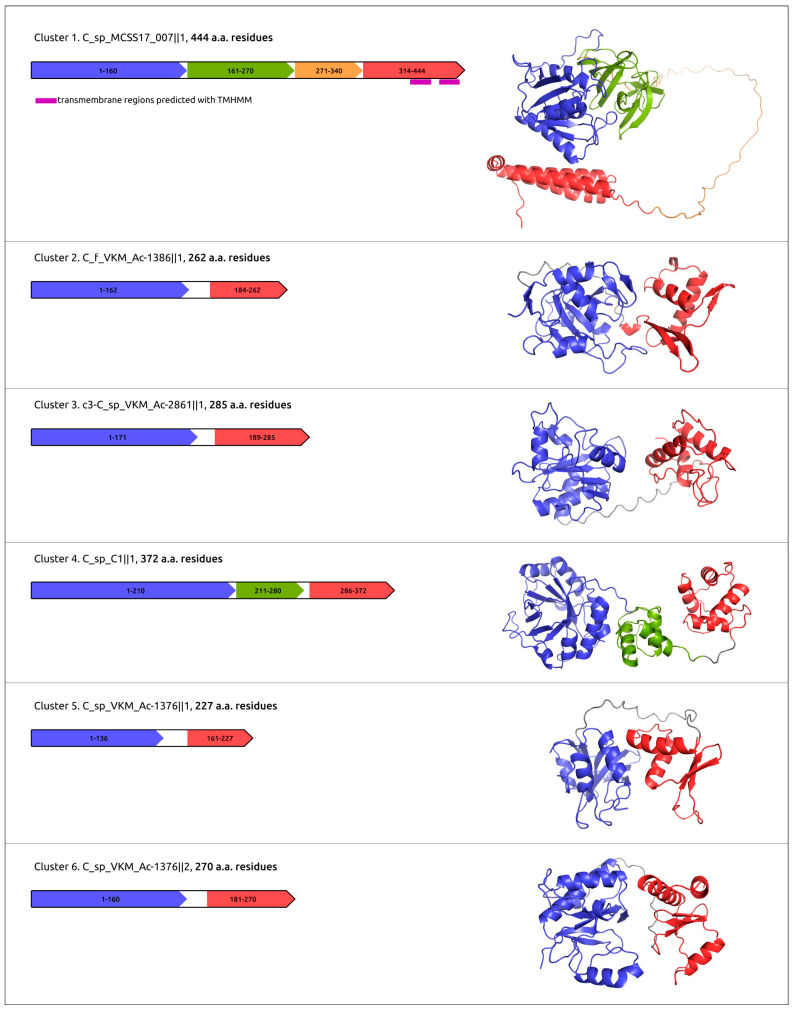
Domain architecture and AlphaFold models of putative endolysins encoded in the putative prophage regions.

A domainal architecture and putative functional assignments of proteins and domains have been suggested using the results of HHpred and InterProScan [62] searches, and clarified using an analysis of the results of protein structural modelling (Figure 8).

**Cluster 1** is represented by a single 444 amino acid residue (aa)-long multidomain endolysin. HHpred HMM-HMM comparisons showed the closeness of the N-terminal domain of this protein (approximately 1–160 aa) to lysins belonging to the γ-glutamyl D,L-endopeptidase (NlpC/P60) family [63]. This domain can be involved with enzymatic activity.

Predicted Domain 2 (161–270 aa) contains putative amino acid residues forming the substrate entrance channel groove [63] and is proposed to be essential for substrate recognition. Domain 3 was not predicted as a compact structure by either AlphaFold 2 [64] or RoseTTAFold [65]. Hypothetically, Domain 3 can facilitate the folding of functional prophage lysin. Domain 4 was predicted to contain transmembrane regions and was modelled to include three α-helices. This domain can assist translocation across the membrane into the peptidoglycan.

Endolysins assigned to **Cluster 2** include eight proteins featuring the two-domain structure with the CHAP catalytic domain putatively arranged in the N-terminal part. It is impossible to predict the catalytic function of these enzymes (amidase or endopeptidase) confidently.

The two endolysins assigned to **Cluster 3** are similar to Cluster 2 lysins. The N-terminal catalytic domain has been proposed to function as N-acetylmuramoyl-L-alanine amidase [66], and the C-terminal domain has appeared to be responsible for peptidoglycan binding [66].

**Cluster 4** contains a single 372-aa-long endolysin with a predicted three-domain architecture. The N-terminal domain is similar to several lytic enzymes of Gram-positive bacteria corresponding to the lysozyme family with muramidase activity cleaving the β-1,4 glycosidic bonds in the backbone of peptidoglycan.

Central and C-terminal domains can facilitate peptidoglycan binding.

**Cluster 5** contains endolysins putatively encoded in 11 PDRs. The N-terminal domains showed a closeness to D-Ala-D-Ala endopeptidases. Structural comparisons of **C_f_VKM_Ac-1386‖1** and *Enterococcus* phage IMEEF1 (Supplementary Figure S7a) showed overall similarity not only to the N-terminal domain, but also to the C-terminal peptidoglycan-binding domain.

**Cluster 6**, the largest cluster, comprises endolysins putatively encoded in 35 PDRs. They are predicted to contain two structural domains. The N-terminal domain is close to the D,D-dipeptidase/D,D-carboxypeptidase proteins. Using a protein structure comparison, Dali [67] indicated the likeness of C-terminal domains of Cluster 6 proteins to the cell-binding domain of *Enterococcus* phage φM1EF22 lysin (RMSD 3.0, PDB code 7D55) [68] (Supplementary Figure S7b).

### 2.7. Analysis of Other Glycopolymer-Degrading Enzymes Encoded in PDRs

**Figure 9:**
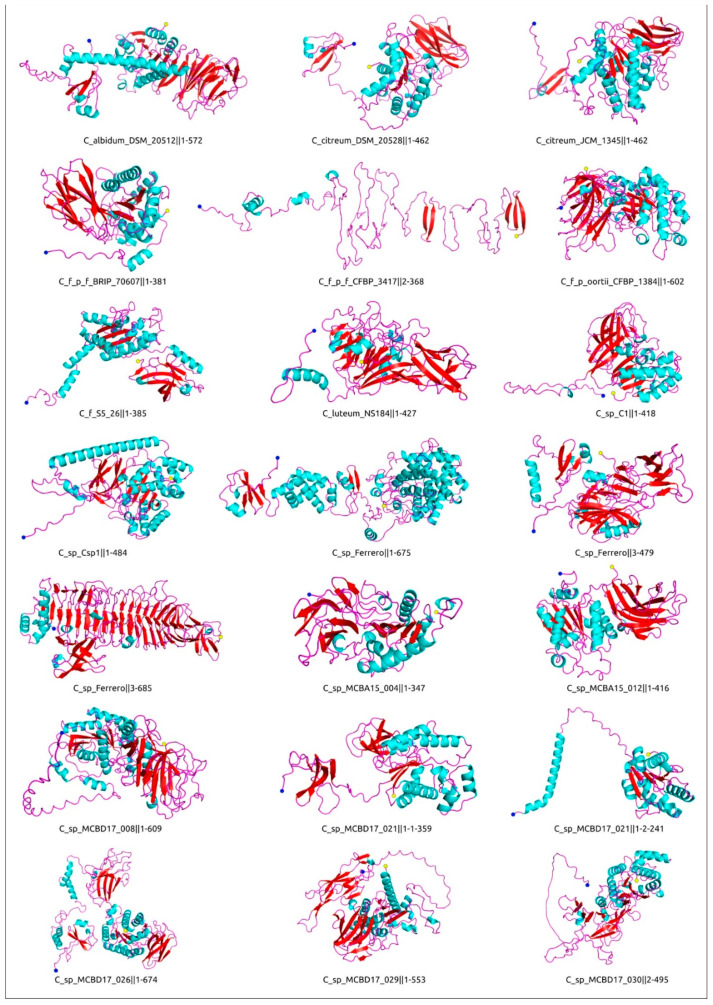
The 21 structural models of depolymerases encoded in the predicted *Curtobacterium* PDRs, coloured by type of secondary structure. The N-terminus is labelled with a blue circle and the C-terminus is labelled with a yellow circle. The remaining models are shown in Supplementary Figure S8.

**Figure 10:**
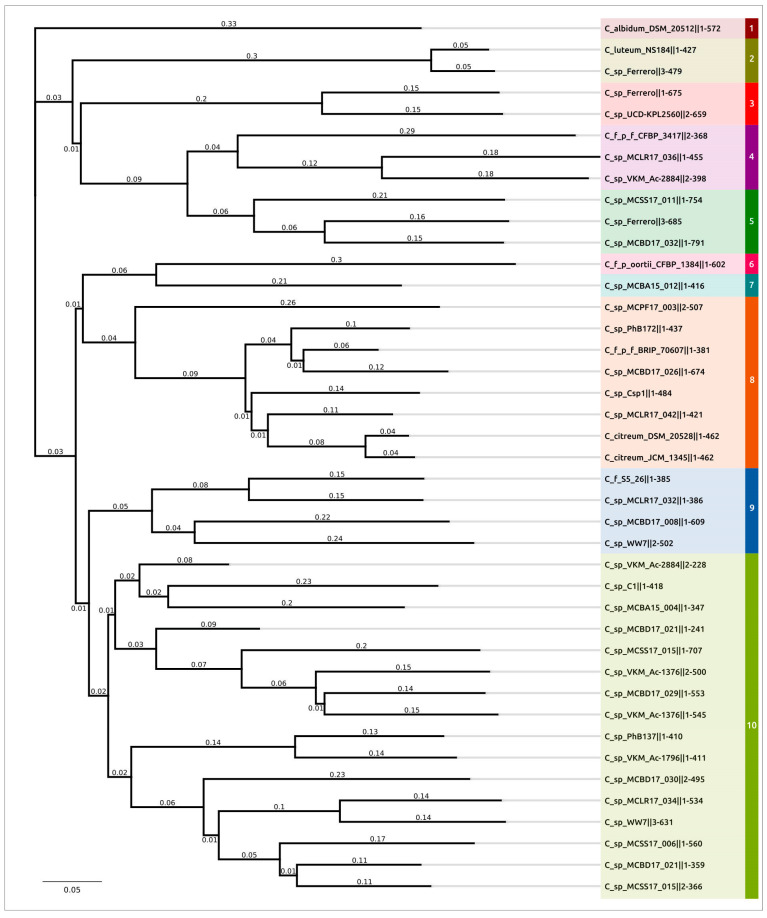
Phylogenetic tree based on amino acid sequences of glycopolymer-degrading enzymes using structural similarity assessed with TM-scores. Branches belonging to different clusters are highlighted using colour. The last three digits in the names of structures indicate the length of protein sequences.

Besides endolysins (peptidoglycan-degrading enzymes), the PDRs analysed contained other genes of putative glycopolymer-degrading enzymes (which, for the purposes of this work, will be referred to as depolymerases, DPOs). These genes are regularly located downstream of the lysis module and can be part of both the lysis system and the penetration apparatus. They were present in the most common putative *Curtobacterium* prophages, in at least 45 of the PDRs analysed (Supplementary File S3). The results of a sequence search and structural analysis indicated a great diversity within the DPOs’ functions and structures (Figure 9, Supplementary Figure S8). In some cases, the phylogenetic analysis using the DPO amino acid sequences resulted in low bootstraps and arranged in common branches the sequences representing the proteins with different enzymatic activity; therefore, clustering using structural similarity was used (Figure 10). This clustering method showed better consistency with the putative functional assignment of DPOs.

**Cluster 1** contains only one α/β-hydrolase (572 aa). The catalytic domain is similar to carboxyl esterase from the oil-degrading bacterium *Oleispira antarctica* (HHpred probability 99.76%, PDB code 3I6Y) [69] and other hydrolases including family S9 peptidases. The catalytic triad Ser^437^-His^549^-Asp^518^ can be easily detected with HHpred and structural alignment using the AF2 model. An enzymatic domain is located in the C-terminal part. The superimposition of **C_albidum_DSM_20512‖1** DPO with carboxyl esterase 3I6Y showed RMSD 2.8 Å (Supplementary Figure S9a).

**Cluster 2** includes two DPOs where an HHpred search indicated a similarity with N-acetylglucosamine-1-phosphodiester α-N-acetylglucosaminidases (NAGPA), which removed the terminal GlcNAc residues [70]. Both DPOs contain three domains including N-terminal α-helical domain and central catalytic domain. The superimposition of **C_luteum_NS184‖1** (427 aa) with NAGPA 6PKU shows RMSD 2.4 Å (Supplementary Figure S9b).

**Cluster 3** comprises two DPOs that are similar to several polysaccharide lyases. Comparison of **C_sp_Ferrero‖1** (675 aa) with the alginate lyase from *Defluviitalea phaphyphila* (PDB code 6JP4) [71] shows a topology that is similar to the C-terminal α/α-barrel domain of 6JP4 and the putative prophage DPO (RMSD 5.9 Å) (Supplementary Figure S12c).

Putative depolymerase domain-containing proteins assigned to **Cluster 4** and **Cluster 5** demonstrated a structural architecture typical of tail fibre (spike) proteins [72,73], including those found in prophage regions [74]. These proteins contained a parallel β-structured pyramidal central part, formed upon trimerisation (Supplementary Figure S10a). The function of such proteins may be assigned as being hyaluronidase, pectate lyase or other enzymes, including the enzymes degrading cell wall components via a lyase mechanism. Analysis of the structures of Cluster 4 DPOs indicated the presence of Asp and Tyr residues located similarly to the well-studied streptococcal phage-encoded hyaluronidase HylP1 [75] (Supplementary Figure S10b). The proteins assigned to Cluster 5 show a similarity with different phage tail spike proteins (TSPs) involved in the degradation of polysaccharides [76,77].

**Cluster 6** contains only one DPO showing a strong similarity with α-L-fucosidase from *Paenibacillus thiaminolyticus* (Supplementary Figure S11a) (HHpred probability about 100.00%, PDB code 6GN6) [78]. This glycoside hydrolase, belonging to the family GH29, cleaves L-fucose from oligosaccharide non-reducing termini.

**Cluster 7** also consists of a single depolymerase similar to GDSL/SGNH hydrolase from *Bacteroides thetaiotaomicron* (probability 99.84%, PDB code 7BR2) [79]. This protein has been suggested to function as an oligosaccharide deacetylase [79]. The topology of the predicated prophage DPO is similar (Supplementary Figure S11b), comprising the N-terminal catalytic domain and C-terminal binding part.

Proteins assigned to Clusters 8, 9 and 10 can also function as polysaccharide deacetylases. **Cluster 8** DPOs show a similarity with the GDSL/SGNH-like lipase/acyl hydrolase family protein from *Neisseria meningitidis* (HHpred probability 99.53%, PDB code 4K7J) (Supplementary Figure S11c). Presumably, the protein functions as an oligosaccharide deacetylase. The central β-barrel domain can function as a carbohydrate-binding part.

**Cluster 9** depolymerases share a similarity with the acyl hydrolase family protein from *Parabacteroides merdae* (PDB code 4Q9A) (Supplementary Figure S11d). The domain architectures of cluster 9 DPOs are basically similar. The structures have three parts: the N-terminal part of variable size includes α-helices, the central part contains the catalytic domain and the C-terminus contains β-strands.

**Cluster 10** comprises proteins with a GDSL/SGNH hydrolase domain and versatile structural organisation. Members of this cluster have a high degree of structural similarity with a group of structurally related proteins, which belong to the SGNH-hydrolase superfamily involved in carbohydrate metabolism and polysaccharide degradation, and which can function as carbohydrate deacetylases.

Most predicted structures contain two domains, where the enzymatic domain is located in the C-terminal part of the molecule; they include the DPOs from **C_sp_MCJR17_043‖1** (553 aa) and identical sequences from the PDRs of other Curtobacterium strains **C_sp_VKM_Ac-1376‖1** (545 aa) (Supplementary Figure S12a), **C_sp_VKM_Ac-1376‖2** (500 aa) (Supplementary Figure S12b), **C_sp_MCBD17_021‖1** (241 aa), **C_sp_MCBA15_004‖1** (347 aa), **C_sp_C1‖1** (418 aa), **C_sp_VKM_Ac-2884‖2** (228 aa) (Supplementary Figure S12c), **C_sp_MCSS17_015‖2** (366 aa), **C_sp_MCBD17_021‖1** (359 aa) and **C_sp_MCBD17_030‖2** (495 aa). Several depolymerases, including those from **C_sp_PhB137‖1** (410 aa), **C_sp_VKM_Ac-1796‖1** (411 aa) (and identical **C_sp_VKM_Ac-2889‖1**), **C_sp_MCLR17_034‖1** (534 aa) and **C_sp_MCSS17_006‖1** (560 aa), have enzymatic domains arranged in the N-terminus.

The C-terminal domains of **C_sp_PhB137‖1** (410 aa) and **C_sp_VKM_Ac-1796‖1** (411 aa) (Supplementary Figure S12d) have similarity with the carbohydrate-binding domain of endo-1,4-beta-xylanase C (PDB code 4XUP). Predicted structures of depolymerase **C_sp_MCSS17_015‖1** (707 aa) (Supplementary Figure S12e) and **C_sp_WW7‖3** (631 aa) feature a more complicated multidomain architecture, where the catalytic domain is located after the N-terminal domain and is attached to the β-barrel subdomain, which in turn is followed by another β-barrel domain. According to the results of an HHpred search, in the case of **C_sp_MCSS17_015‖1** (707 aa), the latter domain can play the role of the additional sugar-binding domain, as in a structurally similar sugar-binding protein (PDB code 4AVS) (Supplementary Figure S12e).

Interestingly, the N-terminal domains of Cluster 10 proteins vary in size and content. Some predicted structures, such as **C_sp_VKM_Ac-1376‖1** (545 aa) and **C_sp_MCSS17_015‖1** (707 aa), contain N-terminal parts composed of β-strands. Hypothetically, such domains could enhance substrate binding. In several models, such as **C_sp_VKM_Ac-1376‖2** (500 aa), the enzymatic domain is arranged between the upstream and downstream sequences, which in turn assemble a compact β-barrel structure, reminiscent of the topology of DPOs assigned to Cluster 8.

## 3. Discussion

Current information on bacteriophages infecting *Curtobacterium* sp. is very sparse. Numerous attempts to isolate lytic phages using traditional techniques [80,81] have resulted in the discovery of just three distinct phage types with prevailing φ29-like podoviruses. The dominance, in the environment, of one, or few, genera of phages infective to a certain bacterial host species has been reported previously. Recent examples relevant to agriculture are the prevalence of *Limestoneviruses* among phages infecting potato pathogen *Dickeya solani* [82,83], and *Ficleduoviruses* among phages of aquaculture pathogen *Flavobacterium columnare* [84]. The accumulation of statistically robust data on available phage diversity either takes decades (as for *E. coli* or *Pseudomonas* sp.), or needs a concerted effort from numerous researchers (as for the SEA-PHAGE programme, studying phages of *Mycobacterium* sp.) [85,86].

A complementary approach is to assess the potential of temperate phages of the target bacteria, including inducible prophages encoded in host genomes. Generally, it is advised that temperate phages are avoided in phage therapy applications. However, when appropriate lytic phages are missing, or temperate phages have unique features, using the latter can be considered [87]. Besides selecting natural *vir* mutants with reduced lysogenic ability, it is possible to improve the behaviour of phages using gene editing approaches [88], or employing recombinant phage-derived enzymes with cytolytic properties [89,90].

Examination of the results of prophage prediction using genomes of different taxonomic groups indicated fewer predicted prophages in *Curtobacterium* bacteria than in most of the other analysed taxa, except for the genus *Clavibacter*, another member of the *Microbacteriaceae* family. This observation is interesting in light of the fact that relatively few *Curtobacterium* strains, (11 of 197 analysed genomes), contain CRISPR-Cas adaptive immune system regions in the search results, while previous research studies have estimated that 50% of sequenced bacterial genomes contain CRISPR [91,92]. It might be suggested that *Curtobacteria* have other effective antiphage defence mechanisms. An investigation of regions related to mobile elements could provide answers to these questions. It is noteworthy that a significant part of the PHASTER prediction results related to genomic regions containing the genes of cell-envelope-modifying enzymes. The cell walls of some *Curtobacterium* strains were shown to contain different glycopolymers, particularly rhamnan, and cell wall hydrolysates contained rhamnose, mannose and other saccharides [93]. Interestingly, the depolymerases assigned to Cluster 5 contained enzymatic domains similar to rhamnogalacturonase and endo-xylogalacturonan.

An analysis of cell-envelope-degrading enzymes of prophage origin might provide insights into the phage resistance mechanisms of *Curtobacteria*. Several prophage depolymerases have been predicted to possess hyaluronidase enzymatic activity. Such proteins have been found in various Gram-positive bacteria, playing an important role in spread and growth [94]. Some phages infecting Gram-positive bacteria use hyaluronidase to break the hyaluronic acid capsule to penetrate the host cell [95,96].

Phages use bacterial receptors to adsorb to the host cell surface. Common cell receptors of Gram-positive bacteria used by phages include murein, cell wall teichoic acids and lipoteichoic acids [97,98]. Bacteria often use modification of the receptors to resist phage infection [99,100]. As part of the host–parasite ‘arms race’, phages evolve to counter the defensive mechanisms of bacteria. Several predicted and modelled phage depolymerases, including the phosphodiester α-N-acetylglucosaminidase (NAGPA) and α-L-fucosidase, can participate in the removal of cell receptors’ modifications, therefore preventing penetration into the host cell.

**Figure 11:**
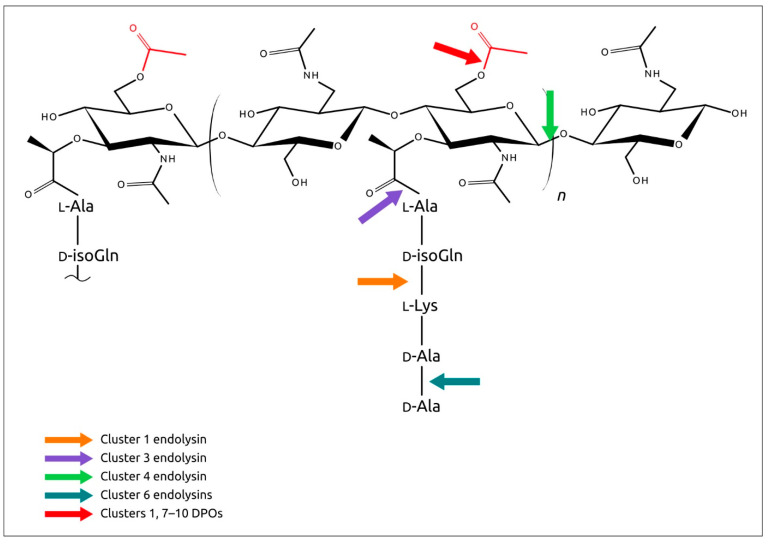
Possible sites for peptidoglycan cleavage, as predicted by cell-wall-degrading enzymes, indicated here with coloured arrows. The O-acetylation of the C-6 OH groups of MurNAc residues is indicated in red.

Most predicted depolymerase could be involved in peptidoglycan (PG) or polysaccharide deacetylation (Figure 11) [55,101,102,103]. O-acetylation of PG occurs at the C-6 hydroxyl of N-acetylmuramoyl residues and sterically blocks the activity of lysozymes [101]. O-acetylation of the capsular polysaccharide is important for bacteria and can achieve polysaccharide rigidity [104,105]. A wide variety of phage cell-envelope-degrading enzymes can indicate their importance as phage counter-defence mechanisms. This, together with the low number of prophages, enables the hypothesis that *Curtobacterium* high phage resistance may be associated with cell wall characteristics. This hypothesis needs further detailed study.

Phage endolysins encoded in predicted prophage-derived regions were represented by several groups showing different types of enzymatic activity, but most of the predicted lysins appeared to exhibit D,D-dipeptidase activity (Figure 11). Predicted structures of predicted endolysins were typical for Gram-positive bacteria, featuring a modular architecture that included at least two domains: catalytic domains and binding domains [106,107,108]. One endolysin, putative γ-D-glutamyl-L-diamino acid endopeptidases from **C_sp_MCSS17_007‖1**, was predicted to have a four-domain architecture, while another endolysin, putative GH25 family muramidase from **C_sp_C1‖1**, was modelled as a three-domain structure. Interestingly, regardless of the number of domains, the catalytic domain was located in the N-terminal part of all proteins. A pronounced modular architecture of endolysins, together with a high level of accuracy of structure predictions using modern AI software, might be used for the design of chimeric proteins that are effective against *Curtobacterium* infections.

## 4. Materials and Methods

### 4.1. Search for Prophage-Derived Sequences

The *Curtobacterium* genomes were downloaded from the NCBI Genome Database [26] and re-annotated using the Prokka pipeline [109], with default settings. The search for possible prophage-derived regions in the genomes was conducted using the PhiSpy pipeline [28] and the PHASTER server [27]. The PhiSpy calculations and PHASTER searches were performed with default settings.

### 4.2. Prophage Annotation

Predicted prophage sequences were extracted using the Geneious Prime 2022.2.1 tools [110] and annotated using Prokka, the HHpred server [32], Phyre 2 [111] and a BLAST search of the NCBI non-redundant (nr/nt) database, as well as a BLAST search of custom databases using GenBank phage sequences. The Prokka settings included using a custom BLAST [31] database built with functionally annotated phage protein sequences extracted from annually annotated PDRs and NCBI GenBank RefSeq database [112] phage genome sequences. The HHpred search results were obtained using the PDB_mmCIF070, SCOPEe70, Pfam-A_v35 and UniProt-SwissProt-viral70 databases. Transmembrane regions were predicted using HHpred and TMHMM [113]. The genetic maps were visualised using Geneious Prime 2022.2.1. Comparisons between the predicted prophage regions were performed and visualised using Easyfig [114], applying the TBLASTX [31] algorithm for the estimation of similarities among genomic loci.

### 4.3. Genomic and Phylogenetic Analysis

Average nucleotide identity was calculated using orthoANI, with default settings [56]. The pairwise nucleotide similarities among the predicted prophage-derived sequences and corresponding similarity matrix were computed using orthoANIu [56] and the Virus Intergenomic Distance Calculator (VIRIDIC) pipeline, with default settings being used [39]. The search for the CRISPR regions was conducted using the MinCED programme [115]. Protein sequence alignments were obtained using Clustal Omega [116] with [number of refinement iterations = 10, evaluate a full distance matrix for initial guide tree and full distance matrix = yes, cluster size for mBed guide trees = 100] settings. The terminase phylogenetic tree was constructed using the RAxML-NG [117] built-in raxmlGUI 2.0.9 graphical user interface [118], using the BLOSUM62+ F+I+G amino acid substitution model [119] and [--bs-metric tbe --tree rand{10} --bs-trees 1000] settings. The best amino acid substitution models were estimated using ModelTest-NG [120]. Robustness of the tree was assessed using a bootstrap analysis employing ten starting trees and 1000 bootstrap replicants, before calculating the transfer bootstrap expectation values. The resulting tree was visualised using the iTOL v6 server [121].

### 4.4. Prophage Induction Assay with Mitomycin C

The induction of prophages was performed as previously described [60], with modifications. Briefly, single colonies of *Curtobacterium* sp. strains VKM Ac-2098, VKM Ac-2884, VKM Ac-2861, VKM Ac-1796, VKM Ac-1376, VKM Ac-2889 and CFBP 3418 were picked from YD-agar (20 g dextrose, 10 g yeast extract, 20 g agar, distilled water up to 1 litre) plates, dropped in tubes containing 10 mL YD-broth (20 g dextrose, 10 g yeast extract, distilled water up to 1 litre) and left to grow overnight at 27 °C in personal bioreactor RTS-1C (Biosan, Riga, Latvia). Overnight bacterial cultures were diluted with 25 mL of fresh YD-broth to OD_600_ of approximately 0,09 and then incubated at 27 °C, with shaking, at 300 rpm for 7 h to obtain a final OD_600_ of 0.25. Then, several aliquots of these bacterial cultures were treated with different concentrations of mitomycin C (0.3 μg/mL, 0.5 μg/mL, 1 μg/mL, 3 μg/mL, 5 μg/mL and 7 μg/mL) or left without mitomycin C, as a control, and incubated under the same conditions for 22 h. After incubation, the samples were centrifuged at 7000 G for 20 min and then passed through 0.45-μm sterile membranes. The resulting filtrates were stored at 4 °C.

The presence of induced phages in the filtrates was tested against the same *Curtobacterium* sp. strains (VKM Ac-2098, VKM Ac-2884, VKM Ac-2861, VKM Ac-1796, VKM Ac-1376, VKM Ac-2889 and CFBP 3418), using the double-layer method [122]. For this, 200 µL of *Curtobacterium* bacterial cultures grown in YD-broth at 27 °C (∼10^8^ CFU per mL) was mixed with 3 mL of soft agar (YD-broth supplemented with 0.7% agar). The mixtures were plated onto the YD-agar. Then, 10 µL of each filtrate was spotted on the soft agar lawns and incubated at 27 °C for 18 to 24 h.

### 4.5. Electron Microscopy

To obtain preparations for microscopy, 100 mL of host culture was grown and prophage was induced, as described above. The resulting lysate was then concentrated and purified, according to the protocol described by Ackermann [123]. Centrifugation with ammonium acetate was carried out twice. Concentrated purified samples were placed on grids and stained with 1% aqueous uranyl acetate (pH 4.0). Prepared grids were examined using a JEM-2100 200 kV transmission electron microscope (JEOL, Tokyo, Japan).

### 4.6. PCR Analysis

PCR primers were constructed with Primer3 2.3.7 [124], using predicted sequences of major capsid proteins. 1-144F (CACCTTCAACGACATCCCCA) and 1-423R (GTAGTTGTCCCAGCCGTTGA) were selected to identify the phage **C_sp_VKM_Ac-2884‖1** (280 bp product). Primers 2-119F (CGTCGCTGTCGTTCAACTTC) and 2-453R (GAAGTCGATCGTCGCCTTGA) were selected to identify the phage **C_sp_VKM_Ac-2884‖2** (335 bp product). 5× ScreenMix (Evrogen, Russia) was used for PCR. Each 25 µL reaction contained 5 µL of ScreenMix, 0.3 µM of each primer and 25 ng of DNA, and the volume was adjusted using sterile Milli-Q water. Thermal cycling conditions were as follows: 94 °C for 3 min, followed by 34 cycles of melting at 94 °C for 30 s, annealing at 60 °C for 30 s, elongation at 30 °C for 30 s and finally incubation at 72 °C for 3 min. As a negative control, a reaction with the addition of an appropriate volume of water was used instead of DNA. PCR results were visualised on a 1.5% agarose gel containing ethidium bromide. For additional verification of the accuracy of determining the site in the genome, Sanger sequencing of the PCR product obtained was carried out.

### 4.7. Computational Modelling and Analysis of Protein Structure

Protein structures were modelled using AlphaFold 2.1, AlphaFold 2.2 [64] and RoseTTAFold, and visualised using Pymol 2.5 (Schrödinger Inc., New York, NY, USA) [125]. The models obtained were superimposed with the experimentally determined structures using Pymol. The robustness of structural alignments was assessed using root-mean-square deviation (RMSD), calculated using Pymol. Multiple protein structure alignment was carried out using mTM-align [126]. The phylogenetic tree was constructed based on the TM-score matrix [127], using the neighbour-joining method [128].

## 5. Conclusions

Due to the prospect of using phages and phage-derived antibacterials for therapy in the context of multi-drug-resistant bacterial infections, genomic studies of prophage-derived regions are of great interest. Studies of *Curtobacterium* genomes have indicated the presence of prophage-derived regions. The number of these regions appears to be smaller than in some other well-studied taxonomic groups, but the analysis and structural modelling of encoded proteins has highlighted the potential of cell-wall-degrading enzymes (CWDEs) for future use. The diversity of CWDEs may indicate the complex structure of the *Curtobacterium* cell envelope, and can facilitate an understanding of the mechanisms of *Curtobacterium* phage resistance.

## Figures and Tables

**Table 1:** General features of genomes of curated, predicted, prophage-derived regions (PDRs).

Group	PDR	Size, kb	GC, %	Bacterial Strain
1	C_sp_C1‖1	47.1	69.5	*Curtobacterium* sp. C1
2	C_sp_MCSS17_015‖2	31.5	67.4	*Curtobacterium* sp. MCSS17_015
3	C_f_S5_26‖1	27.2	68.3	*Curtobacterium flaccumfaciens* S5_26
	C_sp_MCLR17_036‖1	26.6	68.6	*Curtobacterium* sp. MCLR17_036
4	C_sp_MCLR17_032‖1	38.3	62.5	*Curtobacterium* sp. MCLR17_032
5	C_luteum_NS184‖1	22.5	62.3	*Curtobacterium luteum* NS184
	C_sp_VKM_Ac-1376‖2	39.4	64.1	*Curtobacterium* sp. VKM Ac-1376
6	C_sp_MCBD17_030‖2	39.4	65.6	*Curtobacterium* sp. MCBD17_030
7	C_sp_UCD-KPL2560‖2	23.2	66.6	*Curtobacterium* sp. UCD-KPL2560
8	C_sp_MCLR17_034‖1	40	61.3	*Curtobacterium* sp. MCLR17_034
9	C_sp_VKM_Ac-2884‖1	43	69	*Curtobacterium* sp. VKM_Ac-2884
10	C_sp_MCBD17_008‖1	35.7	69.7	*Curtobacterium* sp. MCBD17_008
	C_sp_WW7‖3	34.8	69.7	*Curtobacterium* sp. WW7
11	C_citreum_DSM_20528‖1	36	69.6	*Curtobacterium citreum* DSM 20528
	C_citreum_JCM_1345‖1	35.9	69.6	*Curtobacterium citreum* JCM 1345
	C_f_VKM_Ac-1386‖1	38.3	69.3	*Curtobacterium flaccumfaciens* VKM Ac-1386
	C_sp_Csp1‖1	37.5	70.1	*Curtobacterium* sp. Csp1
	C_sp_Ferrero‖1	34.9	65.4	*Curtobacterium* sp. Ferrero
	C_sp_MCLR17_042‖1	35.5	65.8	*Curtobacterium* sp. MCLR17_042
	C_sp_MCSS17_006‖1	35.6	65.7	*Curtobacterium* sp. MCSS17_006
	C_sp_MCSS17_011‖1	36.7	65.4	*Curtobacterium* sp. MCSS17_011
	C_sp_PhB172‖1	35.9	65.3	*Curtobacterium* sp. PhB172
12	C_f_p_f_BRIP_70607‖1	34.6	65.8	*Curtobacterium flaccumfaciens* pv. *flaccumfaciens* BRIP_70607
	C_f_p_oortii_CFBP_1384‖1	36.3	65.6	*Curtobacterium flaccumfaciens* pv. *oortii* CFBP 1384
	C_sp_MCBD17_028‖1	39.3	69.4	*Curtobacterium* sp. MCBD17_028
	C_sp_PhB137‖1	34.1	65.2	*Curtobacterium* sp. PhB137
13	C_f_p_f_CFBP_3417‖1	17.5	67.8	*Curtobacterium flaccumfaciens* pv. *flaccumfaciens* CFBP 3417
	C_pusillum_ATCC_19096‖1	17	68.5	*Curtobacterium pusillum* ATCC 19096
	C_sp_Csp2‖1	16.8	68.9	*Curtobacterium* sp. Csp2‖1
	C_sp_MCBD17_029‖1	17.2	69.1	*Curtobacterium* sp. MCBD17_029‖1
	C_sp_MCBD17_032‖1	17.7	69.3	*Curtobacterium* sp. MCBD17_032‖1
	C_sp_MCJR17_043‖1	17.2	69.2	*Curtobacterium* sp. MCJR17_043‖1
	C_sp_MCJR17_055‖1	17.2	69.2	*Curtobacterium* sp. MCJR17_055‖1
	C_sp_MCPF17_015‖1	17.2	69.2	*Curtobacterium* sp. MCPF17_015‖1
	C_sp_PhB146‖1	17.8	69.1	*Curtobacterium* sp. PhB146‖1
	C_sp_VKM_Ac-1376‖1	17.2	69.5	*Curtobacterium* sp. VKM Ac-1376
	C_sp_VKM_Ac-2861‖1	17.5	69.2	*Curtobacterium* sp. VKM Ac-2861
14	C_sp_9128‖1	38.4	69.2	*Curtobacterium* sp. 9128
	C_sp_MCBA15_012‖1	41.2	70.8	*Curtobacterium* sp. MCBA15_012
15	C_f_p_f_CFBP_8824‖1	16.5	69.5	*Curtobacterium flaccumfaciens* pv. *flaccumfaciens* CFBP 8824
	C_f_p_oortii_CFBP_1384‖2	16.5	68.3	*Curtobacterium flaccumfaciens* pv. *oortii*_CFBP 1384
	C_sp_MCBA15_008‖1	15.2	64.6	*Curtobacterium* sp. MCBA15_008
	C_sp_MCBD17_035‖1	16.5	66	*Curtobacterium* sp. MCBD17_035
	C_sp_MCBD17_040‖2	16.2	66.5	*Curtobacterium* sp. MCBD17_040
	C_sp_MCSS17_011‖2	16.5	65.3	*Curtobacterium* sp. MCSS17_011
	C_sp_YC1‖2	17.2	65.2	*Curtobacterium* sp. YC1
16	C_sp_MCSS17_007‖1	37.9	62.1	*Curtobacterium* sp. MCSS17_007
17	C_sp_MCBA15_004‖1	46.3	65.4	*Curtobacterium* sp. MCBA15_004
	C_sp_MCBD17_021‖1	47.1	67.8	*Curtobacterium* sp. MCBD17_021
	C_sp_MCSS17_015‖1	45	63.8	*Curtobacterium* sp. MCSS17_015
	C_sp_VKM_Ac-2884‖2	43.9	69.2	*Curtobacterium* sp. VKM Ac-2884
18	C_albidum_DSM_20512‖1	38.2	70.2	*Curtobacterium albidum* DSM 20512
	C_sp_Ferrero‖3	41	70	*Curtobacterium* sp. Ferrero
	C_sp_MCBA15_004‖2	14	69.8	*Curtobacterium* sp. MCBA15_004
19	C_sp_ISL-83‖1	36.7	66.9	*Curtobacterium* sp. ISL-83
	C_sp_MCBD17_003‖1	24.7	68.5	*Curtobacterium* sp. MCBD17_003
20	C_f_p_f_CFBP_3417‖2	37	67.3	*Curtobacterium flaccumfaciens* pv. *flaccumfaciens* CFBP 3417
	C_sp_MCBA15_009‖3	36.8	67.8	*Curtobacterium* sp. MCBA15_009
	C_sp_MCBD17_026‖1	36.8	67	*Curtobacterium* sp. MCBD17_026
	C_sp_MCPF17_003‖2	37.3	68.5	*Curtobacterium* sp. MCPF17_003
	C_sp_PhB25‖1	36.6	69	*Curtobacterium* sp. PhB25
	C_sp_VKM_Ac-1796‖1	37.2	67.3	*Curtobacterium* sp. VKM Ac-1796
	C_sp_VKM_Ac-2889‖1	37.2	67.3	*Curtobacterium* sp. VKM Ac-2889
	C_sp_WW7‖2	36.4	66.6	*Curtobacterium* sp. WW7

## Data Availability

Not applicable.

## Supplementary Materials

The following supporting information can be downloaded at: https://www.mdpi.com/article/10.3390/ijms24021586/s1.
