# Supplementary figures and images for: Prophage-Derived Regions in Curtobacterium Genomes: Good Things, Small Packages
Source: Int J Mol Sci. 2023 Jan 13;24(2):1586. doi: 10.3390/ijms24021586 (PMC9862828; doi:10.3390/ijms24021586)

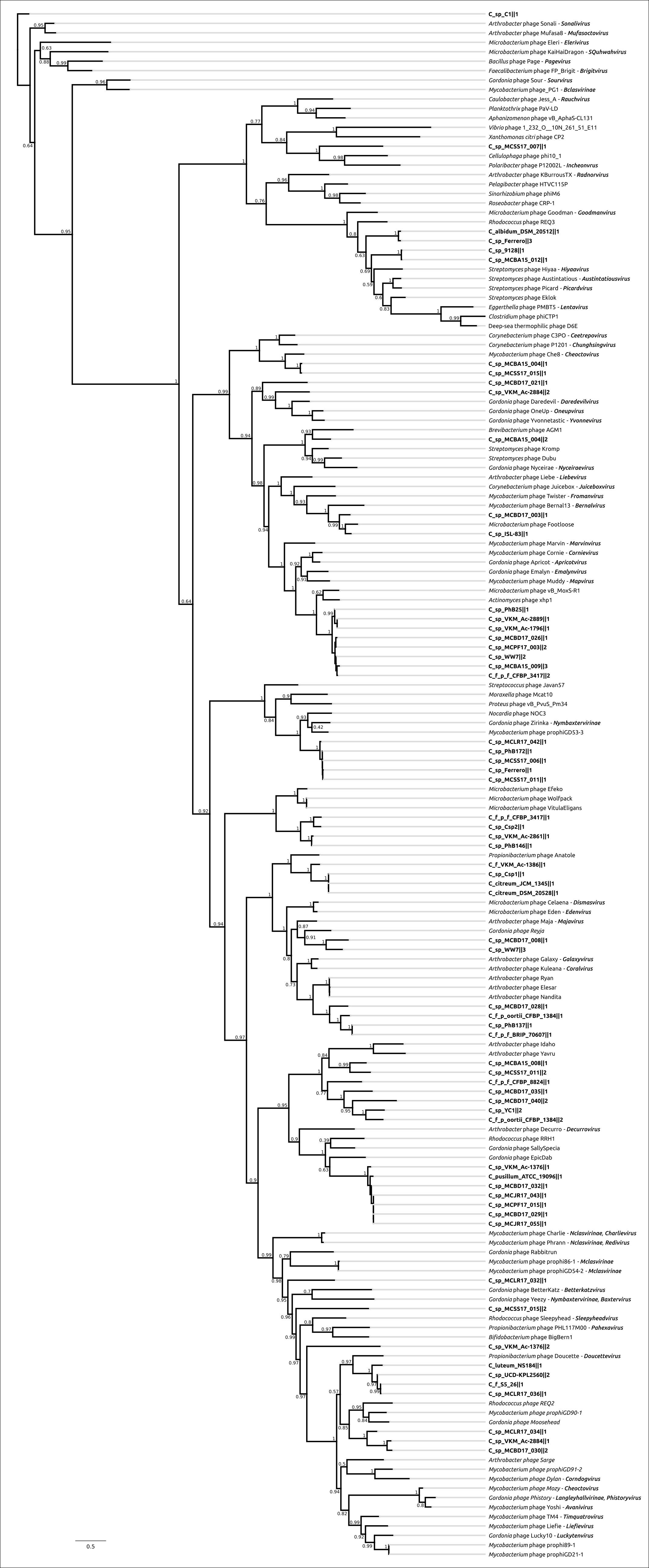

Supplement: Supplementary file 1 [file ijms-24-01586-s001.zip › Figure S1.jpg]

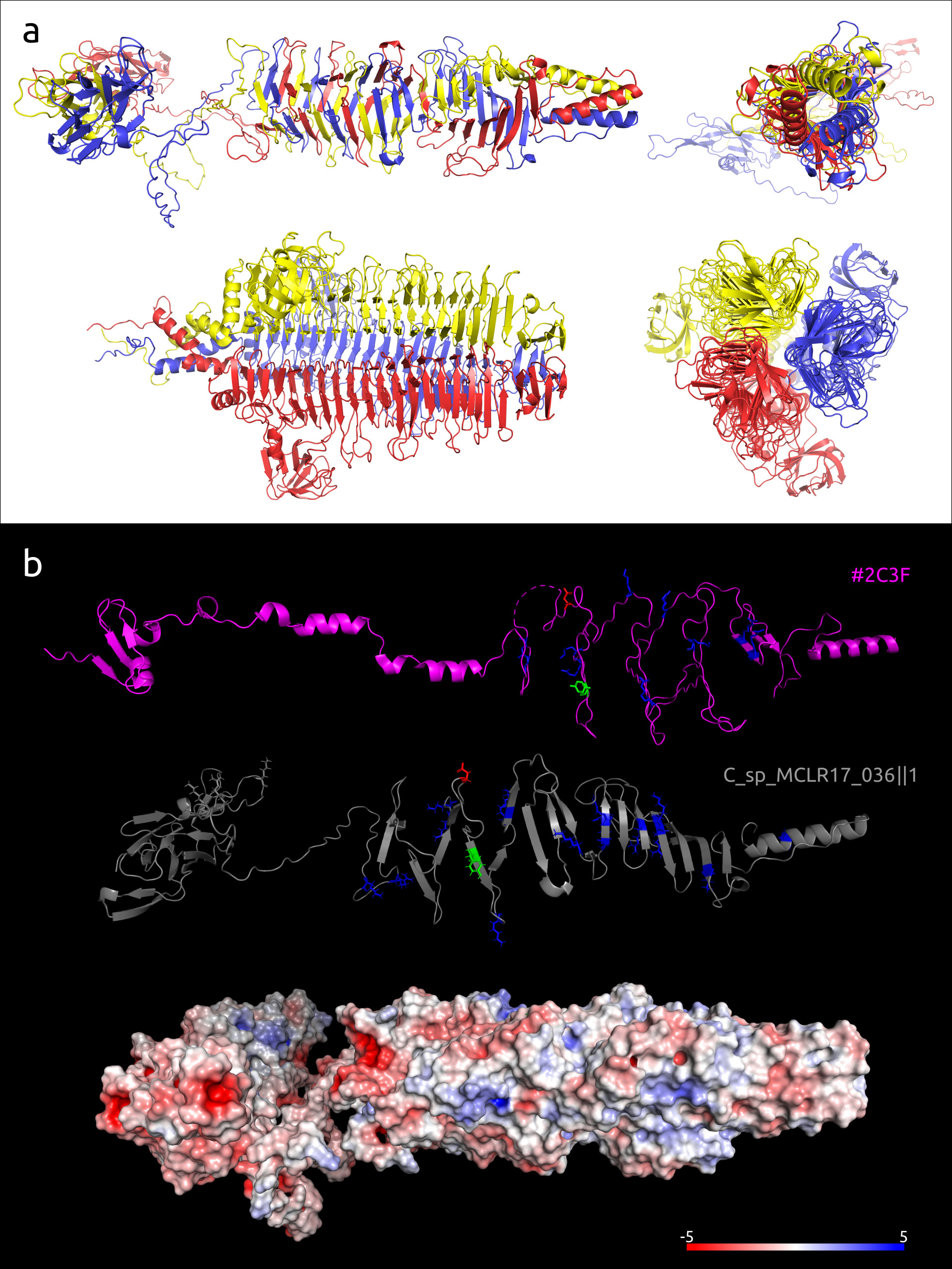

Supplement: Supplementary file 1 [file ijms-24-01586-s001.zip › Figure S10.jpg]

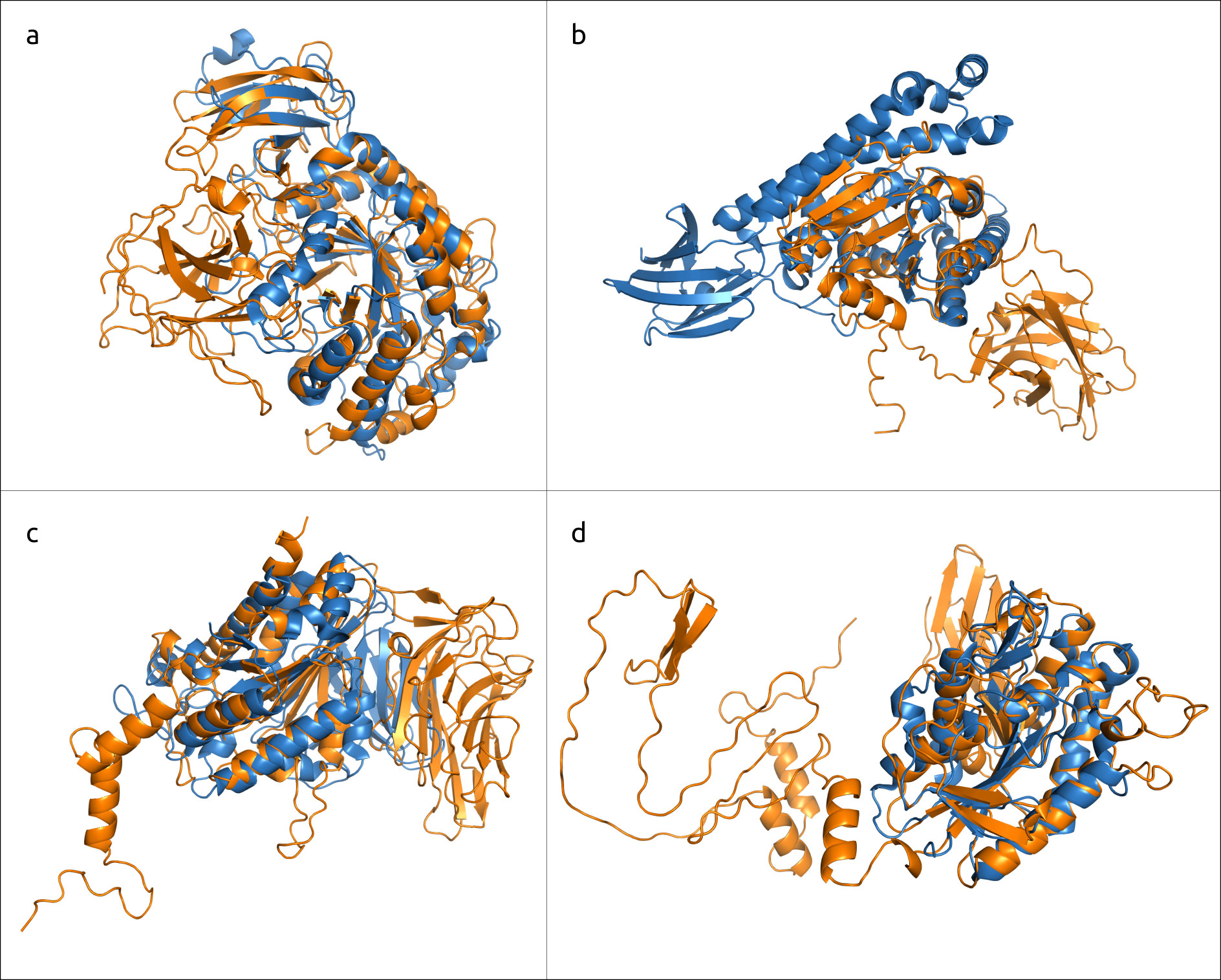

Supplement: Supplementary file 1 [file ijms-24-01586-s001.zip › Figure S11.jpg]

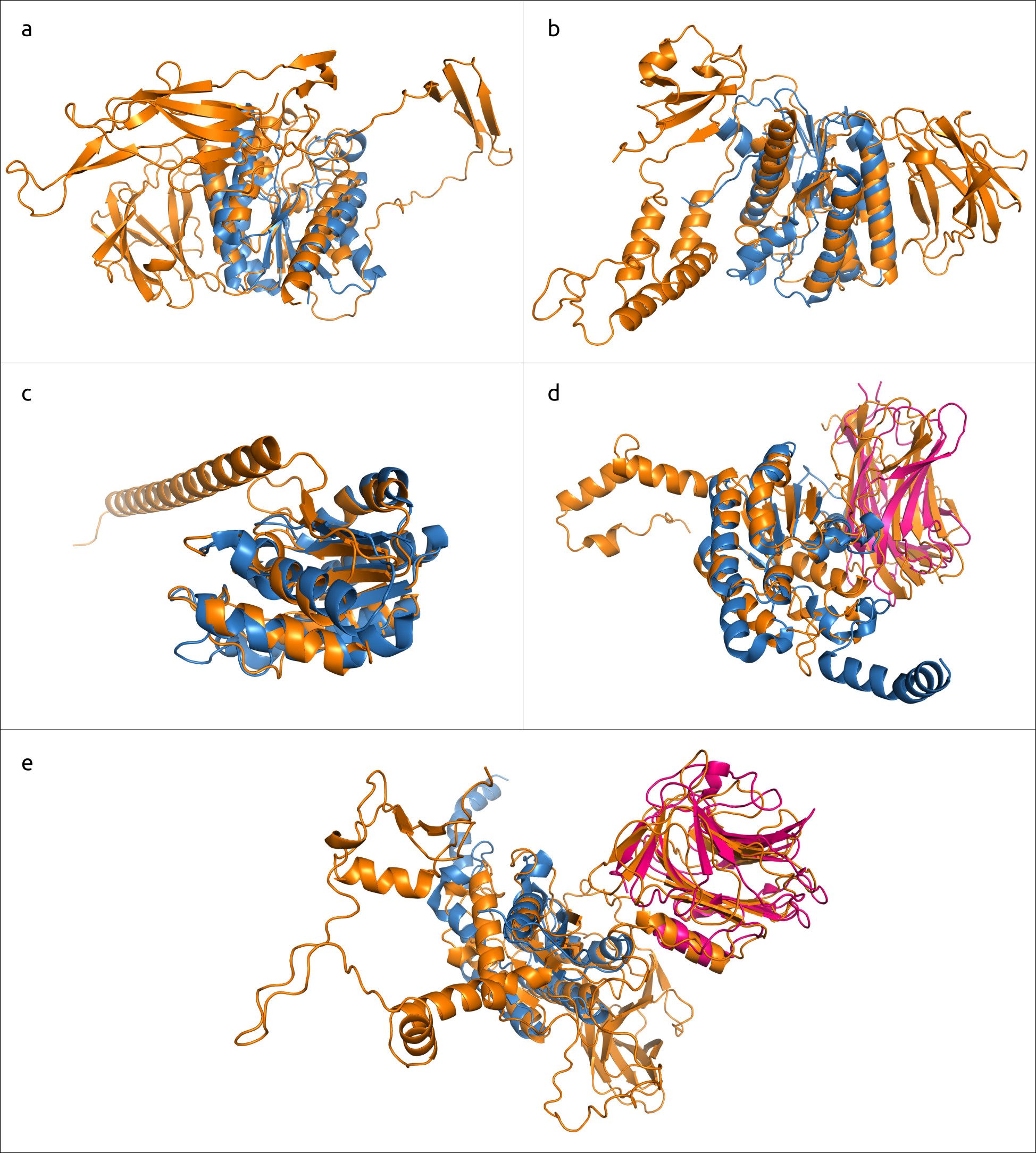

Supplement: Supplementary file 1 [file ijms-24-01586-s001.zip › Figure S12.jpg]

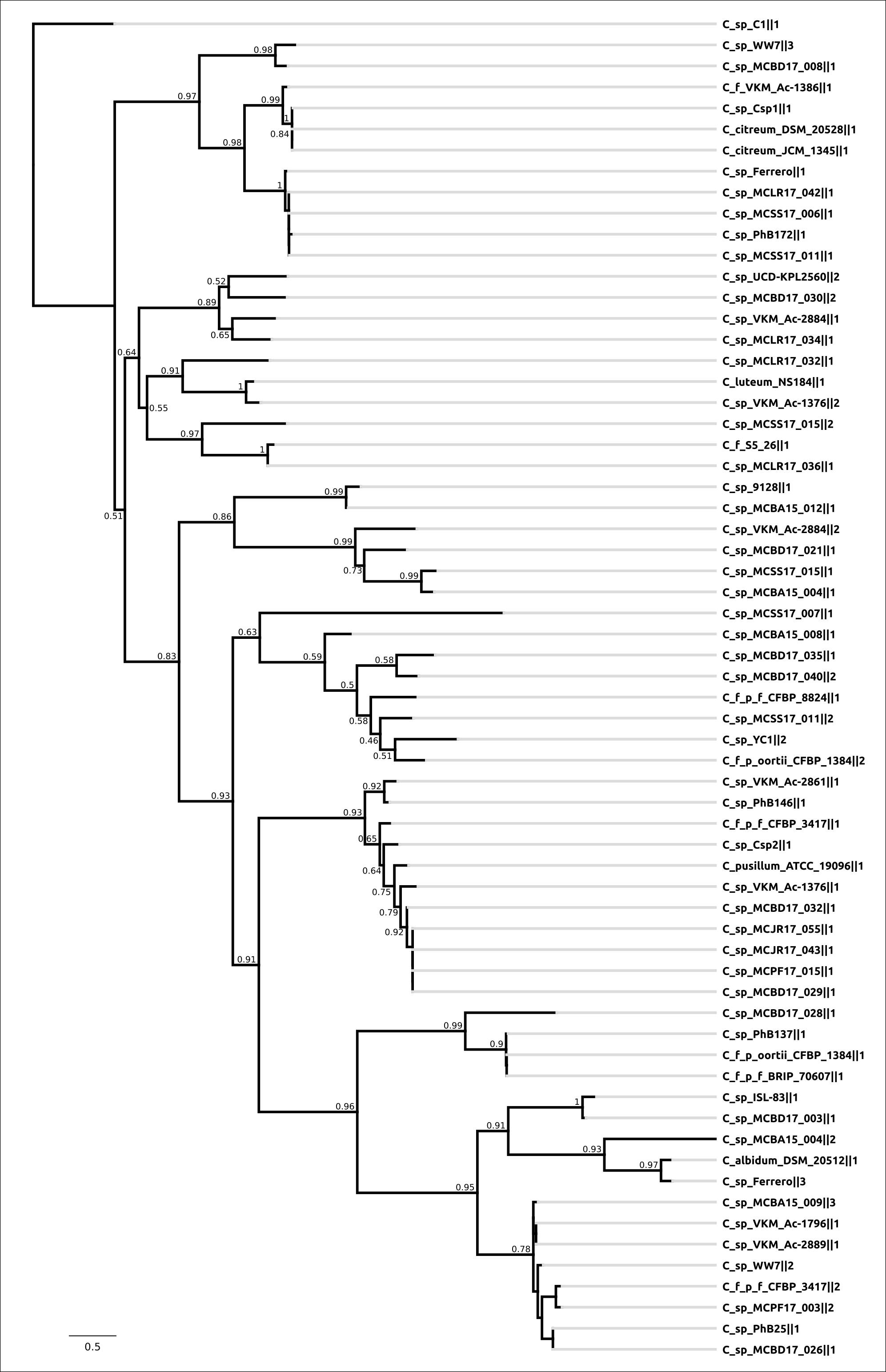

Supplement: Supplementary file 1 [file ijms-24-01586-s001.zip › Figure S2.jpg]

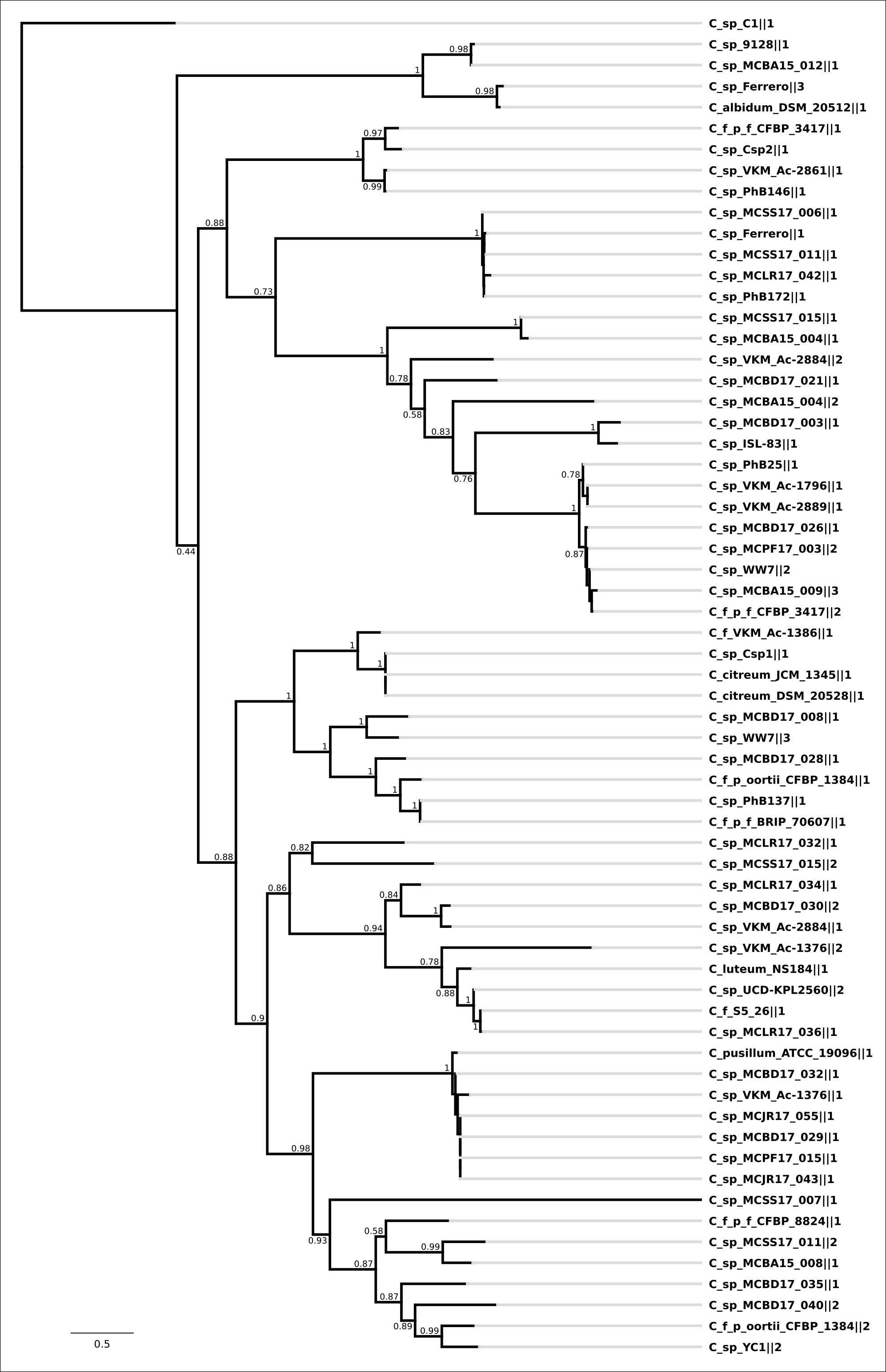

Supplement: Supplementary file 1 [file ijms-24-01586-s001.zip › Figure S3.jpg]

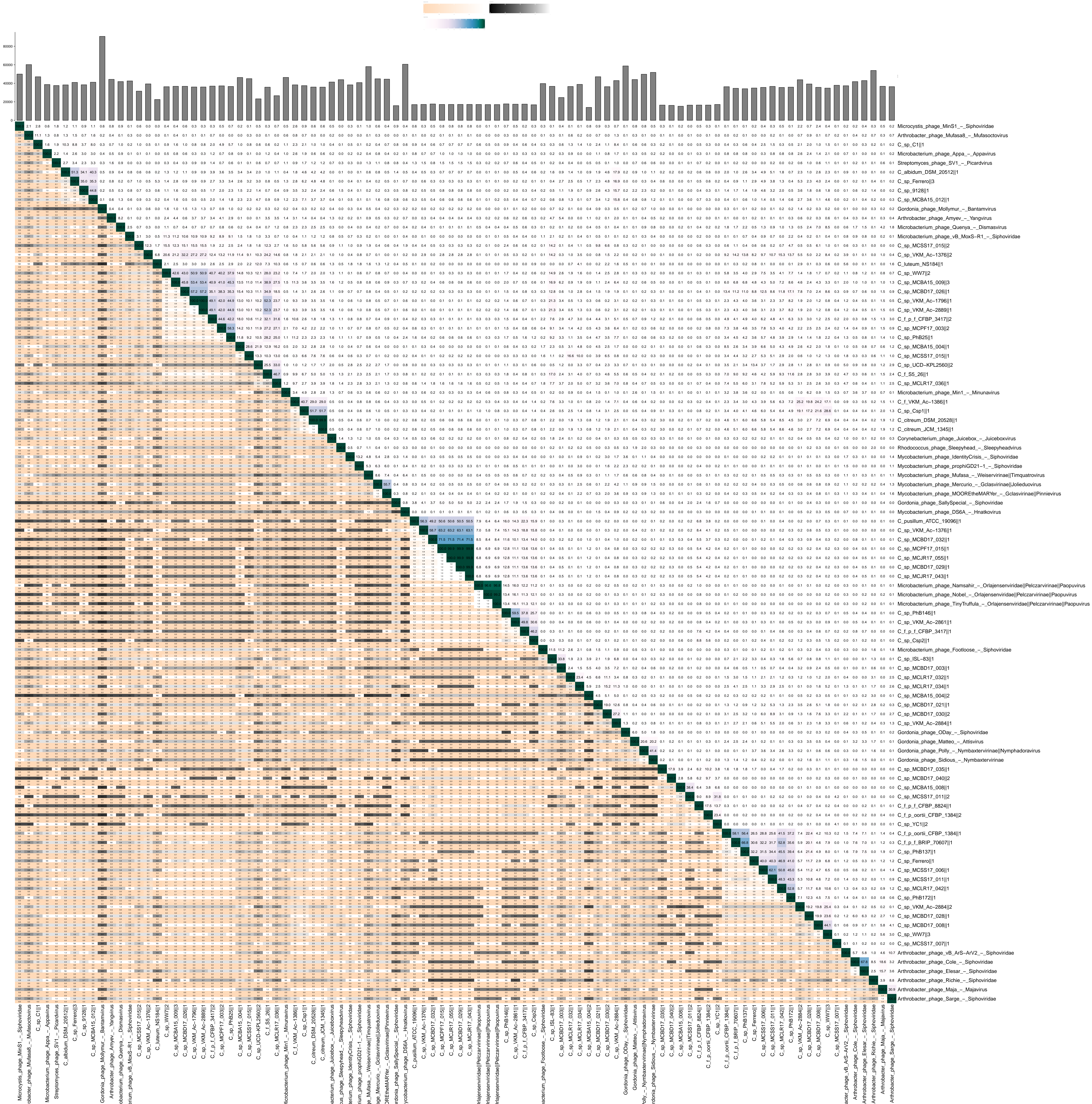

Supplement: Supplementary file 1 [file ijms-24-01586-s001.zip › Figure S4.pdf]

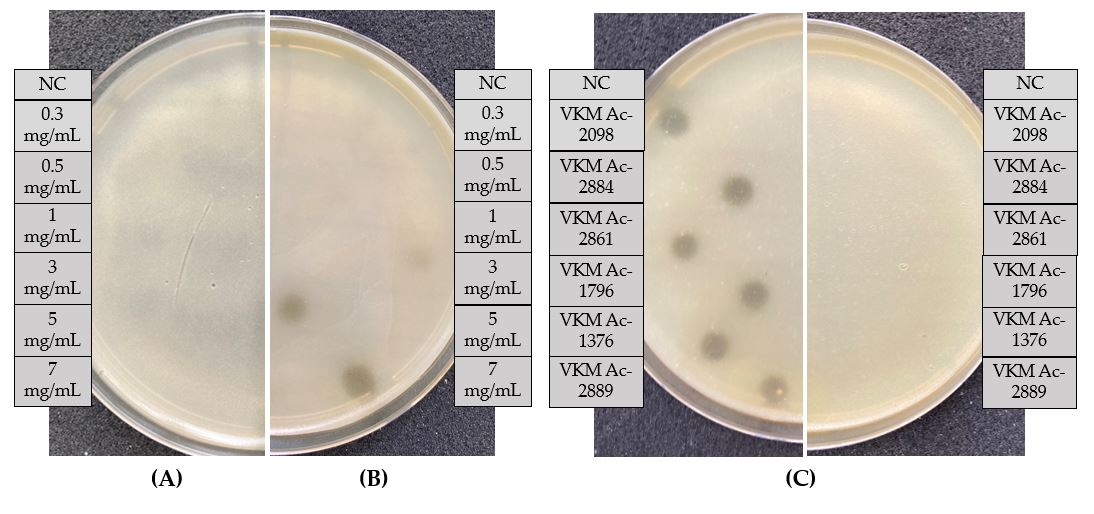

Supplement: Supplementary file 1 [file ijms-24-01586-s001.zip › Figure S5.JPG]

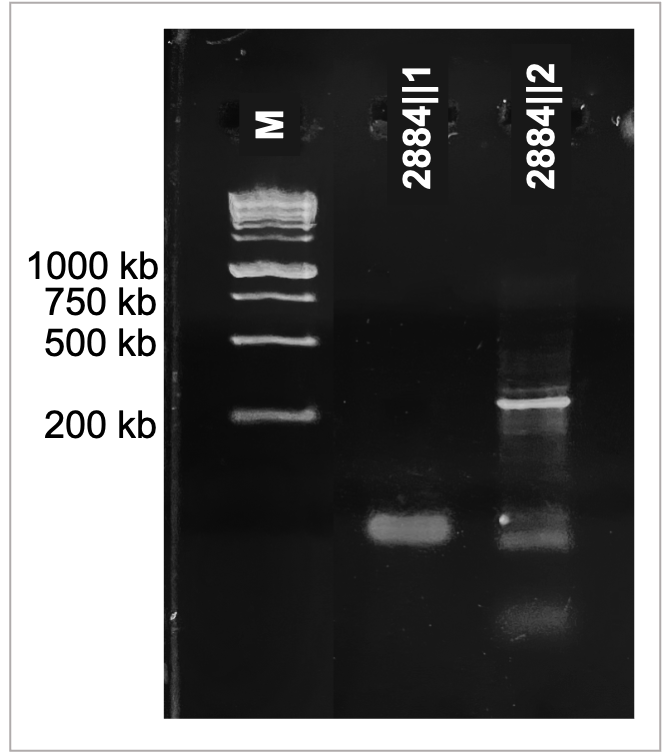

Supplement: Supplementary file 1 [file ijms-24-01586-s001.zip › Figure S6.png]

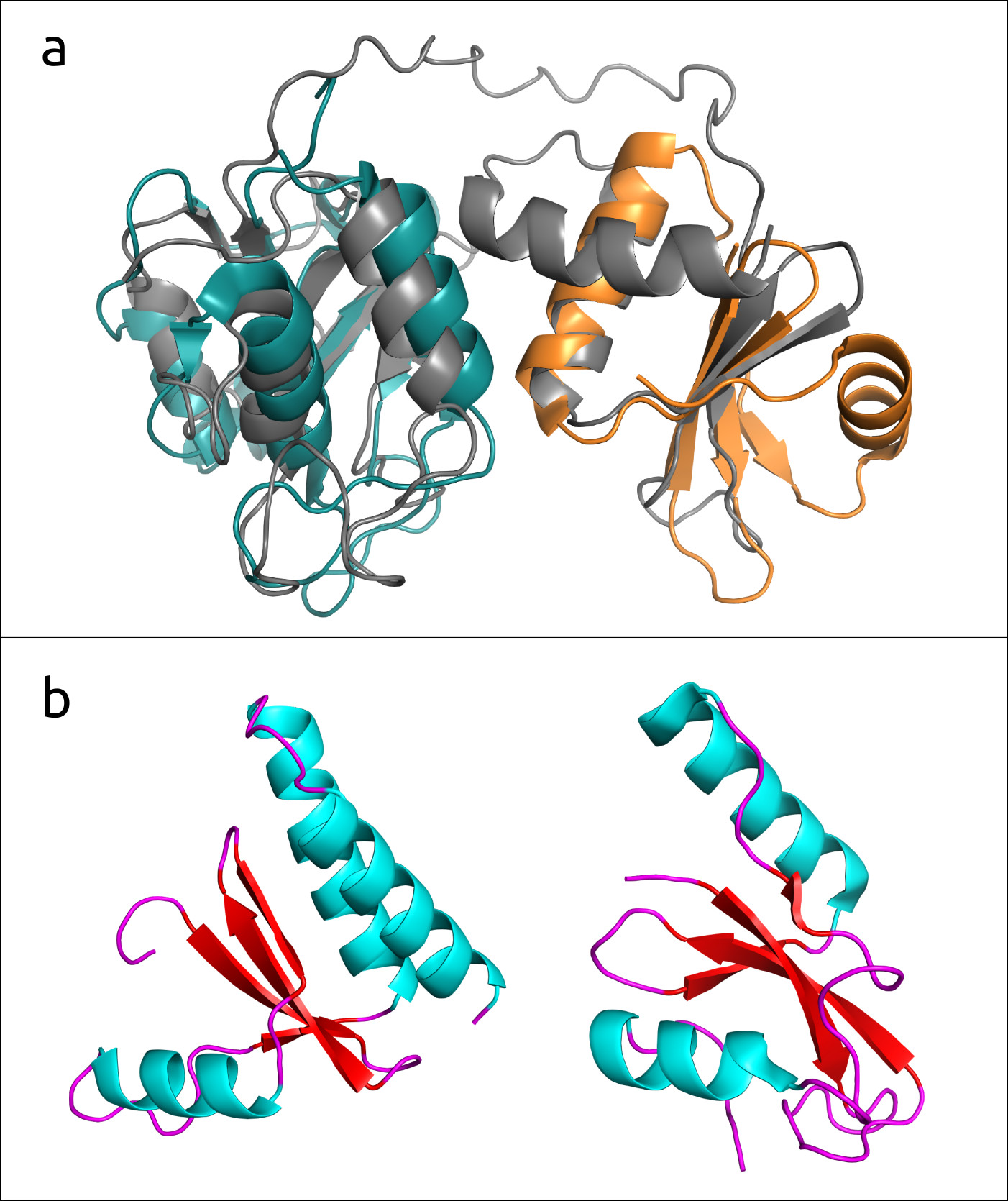

Supplement: Supplementary file 1 [file ijms-24-01586-s001.zip › Figure S7.jpg]

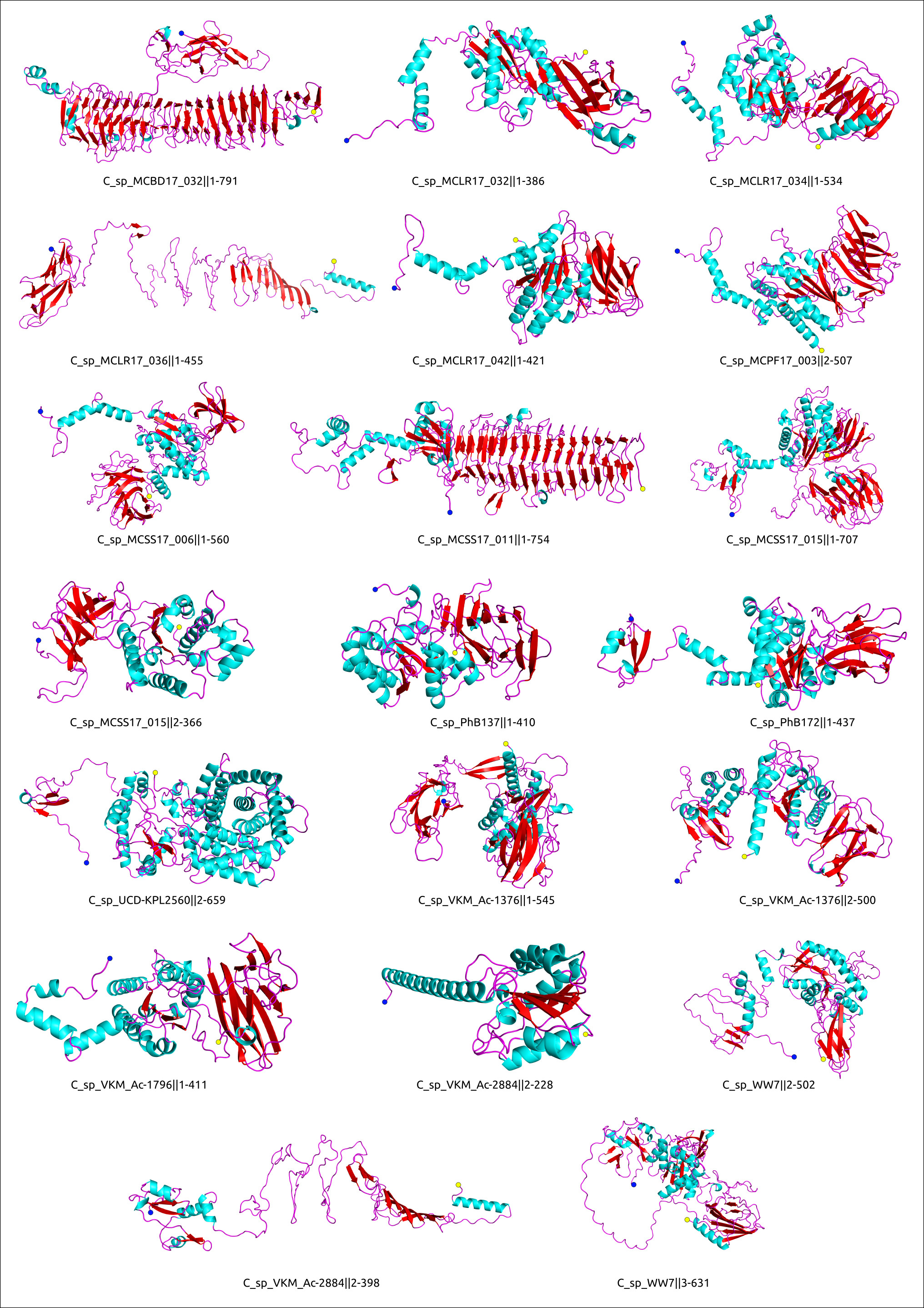

Supplement: Supplementary file 1 [file ijms-24-01586-s001.zip › Figure S8.jpg]

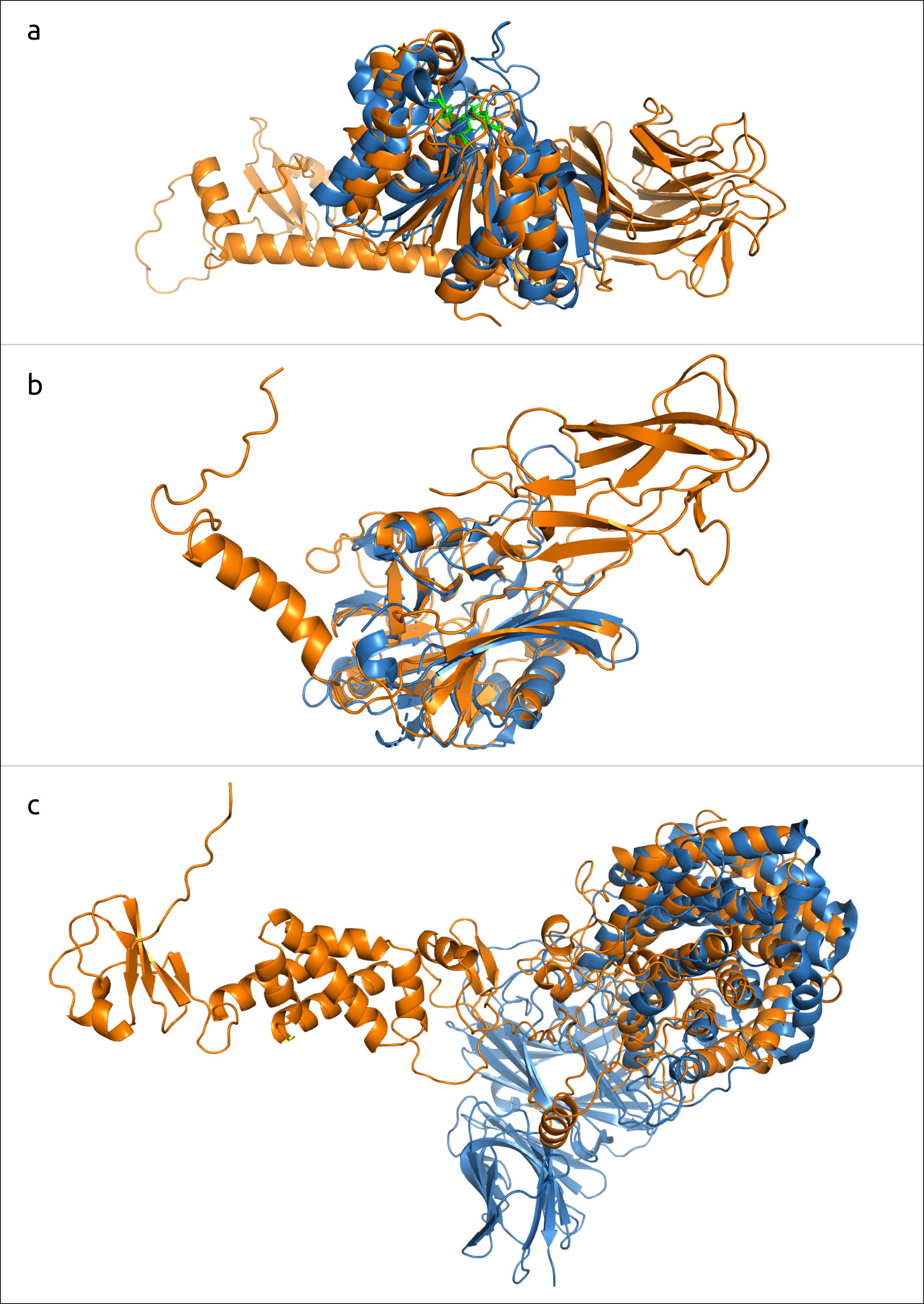

Supplement: Supplementary file 1 [file ijms-24-01586-s001.zip › Figure S9.jpg]
